# Persistent high glucose induced EPB41L4A‐AS1 inhibits glucose uptake via GCN5 mediating crotonylation and acetylation of histones and non‐histones

**DOI:** 10.1002/ctm2.699

**Published:** 2022-02-20

**Authors:** Weijie Liao, Naihan Xu, Haowei Zhang, Weifang Liao, Yanzhi Wang, Songmao Wang, Shikuan Zhang, Yuyang Jiang, Weidong Xie, Yaou Zhang

**Affiliations:** ^1^ State Key Laboratory of Chemical Oncogenomics Tsinghua Shenzhen International Graduate School Tsinghua University Shenzhen P. R. China; ^2^ Key Lab in Healthy Science and Technology Division of Life Science Tsinghua Shenzhen International Graduate School Tsinghua University Shenzhen P. R. China; ^3^ School of Life Sciences Tsinghua University Beijing P. R. China; ^4^ Open FIESTA Center Tsinghua University Shenzhen P. R. China; ^5^ College of life science and technology Wuhan Polytechnic University Wuhan P. R. China

**Keywords:** acetylation, crotonylation, EPB41L4A‐AS1, GCN5, T2DM

## Abstract

**Background:**

Persistent hyperglycemia decreases the sensitivity of insulin‐sensitive organs to insulin, owing to which cells fail to take up and utilize glucose, which exacerbates the progression of type 2 diabetes mellitus (T2DM). lncRNAs' abnormal expression is reported to be associated with the progression of diabetes and plays a significant role in glucose metabolism. Herein, we study the detailed mechanism underlying the functions of lncRNA EPB41L4A‐AS1in T2DM.

**Methods:**

Data from GEO datasets were used to analyze the expression of EPB41L4A‐AS1 between insulin resistance or type 2 diabetes patients and the healthy people. Gene expression was evaluated by qRT‐PCR and western blotting. Glucose uptake was measured by Glucose Uptake Fluorometric Assay Kit. Glucose tolerance of mice was detected by Intraperitoneal glucose tolerance tests. Cell viability was assessed by CCK‐8 assay. The interaction between EPB41L4A‐AS1 and GCN5 was explored by RNA immunoprecipitation, RNA pull‐down and RNA‐FISH combined immunofluorescence. Oxygen consumption rate was tested by Seahorse XF Mito Stress Test.

**Results:**

EPB41L4A‐AS1 was abnormally increased in the liver of patients with T2DM and upregulated in the muscle cells of patients with insulin resistance and in T2DM cell models. The upregulation was associated with increased TP53 expression and reduced glucose uptake. Mechanistically, through interaction with GCN5, EPB41L4A‐AS1 regulated histone H3K27 crotonylation in the GLUT4 promoter region and nonhistone PGC1β acetylation, which inhibited GLUT4 transcription and suppressed glucose uptake by muscle cells. In contrast, EPB41L4A‐AS1 binding to GCN5 enhanced H3K27 and H3K14 acetylation in the TXNIP promoter region, which activated transcription by promoting the recruitment of the transcriptional activator MLXIP. This enhanced GLUT4/2 endocytosis and further suppressed glucose uptake.

**Conclusion:**

Our study first showed that the EPB41L4A‐AS1/GCN5 complex repressed glucose uptake via targeting GLUT4/2 and TXNIP by regulating histone and nonhistone acetylation or crotonylation. Since a weaker glucose uptake ability is one of the major clinical features of T2DM, the inhibition of EPB41L4A‐AS1 expression seems to be a potentially effective strategy for drug development in T2DM treatment.

## Graphical abstract

1. LncRNA EPB41L4A‐AS1 was abnormally increased in the liver of T2DM patients, muscle of insulin resistance patients and T2DM cell models.
2. LncRNA EPB41L4A‐AS1 was interacted with GCN5 and inhibited GLUT4 transcription via regulating H3K27cr and acetylation of PGC1‐β.
3. LncRNA EPB41L4A‐AS1 decreased glucose uptake by enhancing GLUT2/4 endocytosis through GCN5‐ mediated acetylation of H3K27 and H3K14 in TXNIP promoter region.

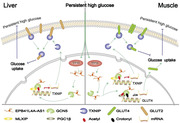

## 1 INTRODUCTION

The incidence of type 2 diabetes mellitus (T2DM) has increased drastically in recent times, and T2DM has become a global health concern^1^ and is expected to constitute a major cause of human death by 2030.^2^ Among several proven pathogeneses, insulin resistance is the earliest pathogenic change observed in T2DM,^3^ the most prominent feature of which is the failure of cells to respond appropriately to insulin secretion, which decreases their glucose uptake ability.^4^, ^5^ The liver and muscles are the primary insulin‐sensitive organs that play important roles in glucose homeostasis.^6^ The liver consumes more than half of the oral glucose intake, and insulin resistance in the liver precedes peripheral insulin resistance such as that in muscles.^7^, ^8^ Muscle cells primarily regulate blood glucose levels via insulin‐sensitive glucose transport such as that occurring via GLUT4/SLC2A4.^9^ The impairment of insulin sensitivity in hepatocytes or muscle cells may disturb the homeostasis of blood glucose concentration, thereby reducing glucose uptake and aggravating T2DM.^10^ Therefore, improvement of the glucose uptake ability in response to insulin seems to be an effective method for treating T2DM.

Long non‐coding RNAs (lncRNAs) participate in several cellular processes including gene transcription and translation and chromatin structure regulation.^11^, ^12^ The abnormal expression of lncRNAs can lead to multiple serious diseases including cardiovascular injury, Alzheimer's disease and cancer.^13^, ^14^, ^15^, ^16^, ^17^, ^18^, ^19^, ^20^, ^21^, ^22^ Increasing evidence shows that lncRNAs’ abnormal expression is also related to the progression of diabetes and plays a significant role in glucose metabolism. For instance, the increase in lncRNA IGF2‐AS expression mediated by high glucose levels is an important contributing factor in the abnormal insulin signaling pathway.^23^ Moreover, lncRNA H19 is down‐regulated in GDM mice, resulting in the impaired glucose uptake ability. H19 primarily regulates glucose metabolism by influencing the methylation of IGF2 DNA.^24^ LncFHL, a fasting‐induced human‐specific and liver‐enriched lncRNA, regulates the fasting plasma glucose levels via the PPAR‐α pathway by interacting with HuR in liver‐specific humanized mice.^25^ lncLGR regulates glycogen storage and glucokinase expression during fasting by suppressing GCK expression.^26^ lncSHGL can attenuate hyperglycaemia and suppress gluconeogenesis by recruiting hnRNPA1 to enhance the translation of CaM in hepatocytes.^27^ These studies indicate that lncRNAs are potentially important regulators of T2DM pathogenesis. However, owing to the vast number of lncRNAs, the detailed mechanism underlying the functions of lncRNAs in T2DM pathogenesis remains unclear and requires further investigation.

EPB41L4A‐AS1 is a recently discovered lncRNA. Previously, we reported that EPB41L4A‐AS1 significantly influenced cancer cell metabolism and caused an abnormal reduction in the energy metabolism.^28^ These results indicate that EPB41L4A‐AS1 could also mediate glucose metabolism in diabetes. In this study, EPB41L4A‐AS1 was up‐regulated in the liver and muscle tissues of patients with T2DM (based on data obtained from specific databases) and cell models of T2DM via a persistent high glucose concentration‐induced increase in TP53 expression, which further inhibited glucose uptake and mitochondrial respiration. Mechanistically, through its interaction with GCN5/lysine acetyltransferase 2A, a histone acetyltransferase and succinyltransferase,^29^ EPB41L4A‐AS1 epigenetically regulated the transcription of GLUT4 and TXNIP by affecting the crotonylation or acetylation of histone 3 at K27 and K14 sites. The loss of function of EPB41L4A‐AS1 improved glucose uptake and enhanced mitochondrial respiration. Our study provides a new insight into T2DM pathomechanisms and suggests a potential target for T2DM treatment.

## 2 RESULTS

### 2.1 EPB41L4A‐AS1 expression was increased in patients with T2DM and T2DM cell models

**FIGURE 1:**
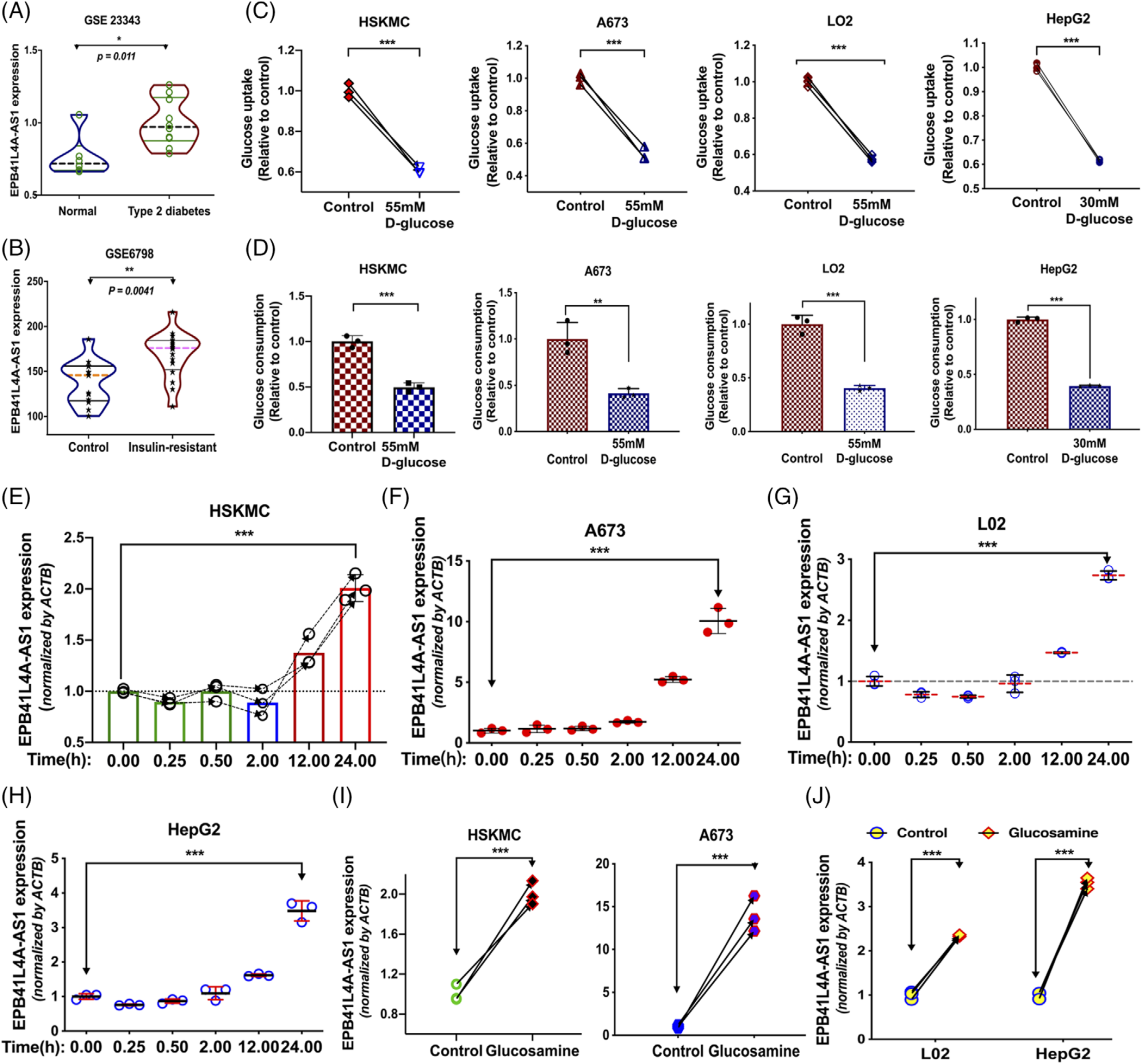
EPB41L4A‐AS1 is up‐regulated in patients with type 2 diabetes mellitus (T2DM) and T2DM cell models. (A‐B) The expression of EPB41L4A‐AS1 in the liver (n = 10) and muscle (n = 16) tissues of patients with T2DM or insulin resistance compared with that in normal controls. Data are from the GEO databases GSE23343 and GSE6798 datasets, Mann–Whitney U test. (C) Glucose uptake in cells before and after treatment at high glucose concentrations (n = 3). (D) Glucose consumption in cells treated at normal glucose concentrations for 6 h after pre‐treatment with high‐ or normal‐glucose DMEM (n = 3). (E‐H) EPB41L4A‐AS1 was measured in cells treated at high glucose concentrations for 0, 0.25, 0.5, 2, 12 and 24 h (n = 3). One‐way analysis of variance, followed by Tukey's post‐hoc test. (I‐J) The expression of EPB41L4A‐AS1 in cells incubated with 5 mM glucosamine for 24 h (n = 3)

To study whether EPB41L4A‐AS1 contributes to T2DM, through gene expression analysis of the GSE23343^30^ and GSE6798^31^ datasets obtained from the Gene Expression Omnibus (GEO) database, EPB41L4A‐AS1 expression was found to be abnormally increased in the liver of patients with T2DM and up‐regulated in the muscle cells of patients with insulin resistance (Figure 1A and B). Among the different clinic‐pathological features of T2DM, insulin resistance is the most significant and can be induced by persistently high glucose or glucosamine levels.^32^, ^33^ To establish T2DM cell models, we used human primary skeletal muscle cells (HSkMC), human muscle source carcinoma cells (A673), human hepatic carcinoma cells (HepG2) and non‐tumor liver cells (L02). The cells were incubated with glucose at high concentrations for 24 h and subjected to glucose uptake assays and glucose consumption assays. The glucose uptake ability of cells incubated in media with persistently high glucose levels was significantly decreased in response to insulin, as demonstrated by the decreased glucose consumption (Figure 1C and D). In addition, the cell counts remained unchanged during the 24 h incubation period (Supporting information Figure S1A‐D). To study the function of EPB41L4A‐AS1 in this process, we measured whether it was abnormally expressed in the four T2DM cell models, of which results showed that EPB41L4A‐AS1 was markedly induced in the models (Figure 1E‐H). Conversely, we also treated these four cell lines with glucosamine, an insulin‐resistance inducer, for 24 h and observed EPB41L4A‐AS1 up‐regulation (Figure 1I and J). These data demonstrated that lncRNA EPB41L4A‐AS1 over‐expression induced by persistently high glucose or glucosamine levels was associated with the inhibition of glucose uptake in T2DM cell models.

### 2.2 High glucose or glucosamine levels enhance EPB41L4A‐AS1 expression by increasing TP53 expression

**FIGURE 2:**
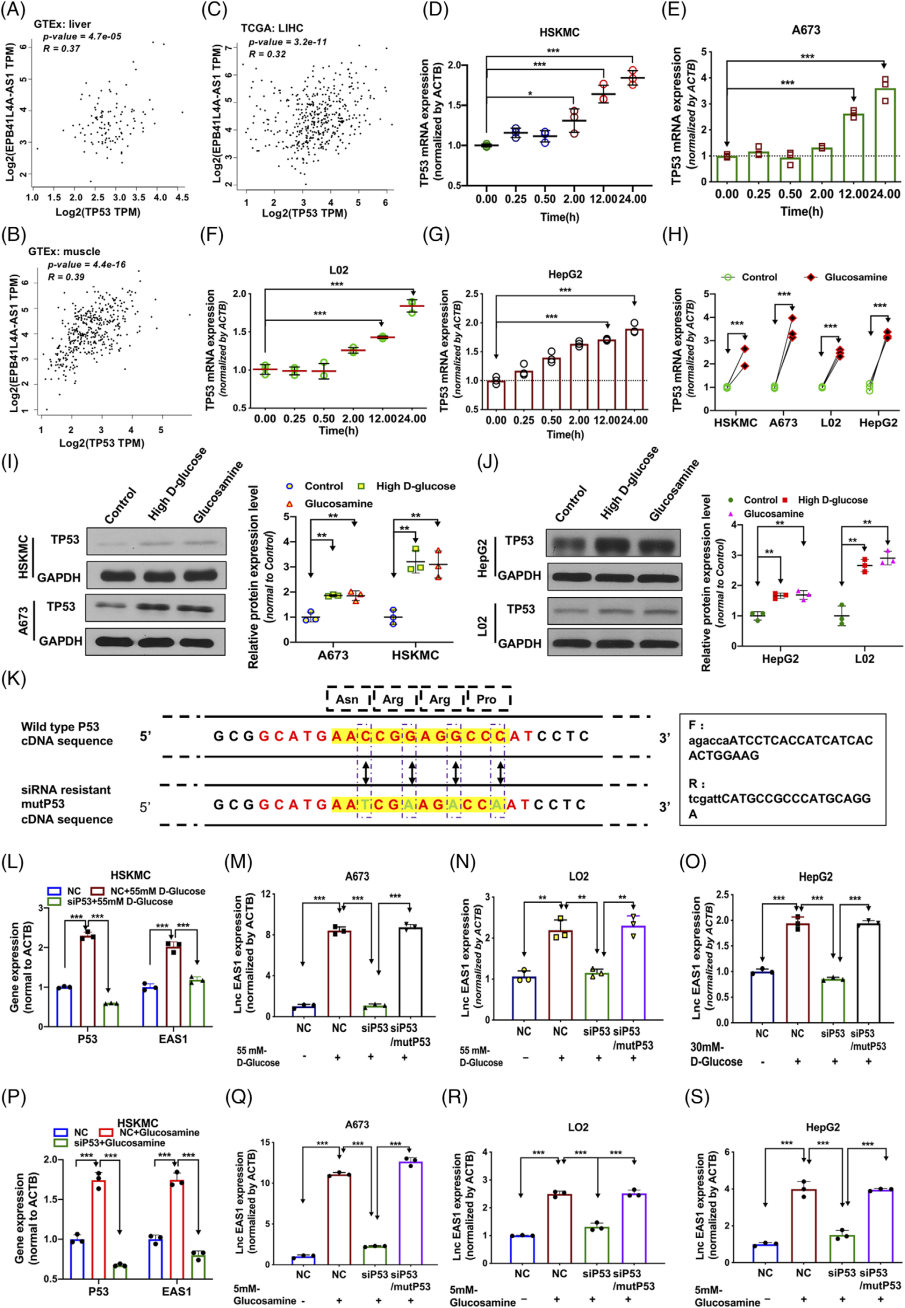
High glucose or glucosamine concentration up‐regulates the expression of EPB41L4A‐AS1 by enhancing TP53 expression. As shown, EAS1 represents EPB41L4A‐AS1; (A‐C) Pearson correlation analysis between EPB41L4A‐AS1 and TP53 expression in human liver and muscle tissues based on data from the GTEx database, and Pearson's correlation analysis of EPB41L4A‐AS1 and TP53 expression based on liver cancer data from the TCGA database. Images were obtained from the GEPIA website. (D‐G) TP53 expression was measured in cells treated at high glucose concentrations for 0, 0.25, 0.5, 2, 12 and 24 h (n = 3). (H) TP53 expression in cells incubated with 5 mM glucosamine; One‐way analysis of variance, followed by Tukey's post‐hoc test. (I‐J) TP53 expression was measured in cells treated at high glucose concentrations or with 5 mM glucosamine. (K) Diagram showing four mutation sites in the siRNA‐target sequence of P53 cDNA without a change in the amino‐acid sequence (mutP53), which could confer resistance to the P53 siRNA used in this study. (L‐O) EPB41L4A‐AS1 expression was analyzed in cells treated at high glucose concentrations for 24 h after transfection with NC, siP53 or siP53, and the mutP53 plasmid for 24 h (n = 3). (P‐S) The RNA expression of EPB41L4A‐AS1 was analyzed using cells treated with 5 mM glucosamine for 24 h after transfection with NC, siP53 or siP53, and the mutP53 plasmid for 24 h (n = 3)

As shown in our previous studies, EPB41L4A‐AS1 down‐regulation is regulated by TP53 in human tumor cells.^28^ Several studies have also shown that TP53 is over‐expressed in the tissues of patients with T2DM with insulin resistance or in insulin‐resistant cells/animal models.^34^, ^35^, ^36^, ^37^ We examined whether the up‐regulation of EPB41L4A‐AS1 was related to an increase in TP53 expression induced by high glucose or glucosamine levels. First, correlation analysis of EPB41L4A‐AS1 and TP53 expression was performed using data for normal human liver and skeletal muscles obtained from the GTEx database, and the results showed positive correlation (liver: *r* = 0.37*, p* value = 4.7e‐05; muscle: *r* *= 0.39, p* value = 3.2e‐11, Figure 2A and B).^38^ Moreover, liver cancer data were also analyzed, and the results indicated a positive correlation (*Pearson's r* = 0.32*, p *< .001, Figure 2C). At cellular level, we measured the mRNA expression of TP53 in the four T2DM cell models under high glucose concentrations and found that TP53 expression was increased (Figure 2D‐G). The same results were observed in cells incubated with glucosamine for 24 h (Figure 2H). In addition, the pattern of changes observed in the mRNA levels was also observed in the TP53 protein levels in the T2DM cell models (Figure 2I and J, Supporting information Figure S2A and B). To further understand the regulatory relationship between TP53 and EPB41L4A‐AS1, TP53‐specific siRNA, control siRNA (negative control, NC) and P53‐specific siRNA‐resistance mutP53 plasmid (Figure 2K) were transfected into the cells, and the cells were then incubated in a medium with high glucose concentration. EPB41L4A‐AS1 up‐regulation induced by the high glucose concentration was significantly suppressed in cells transfected with TP53 siRNA (Figure 2L‐O, Supporting information Figure S2C‐E), but was rescued by mutP53 over‐expression (Figure 2M‐O). Consistent with the trend of changes induced by high glucose concentrations, the increase in EPB1L4A‐AS1 expression induced by glucosamine was nearly abolished in response to TP53 inhibition (Figure 2P‐S), but was rescued by mutP53 over‐expression (Figure 2Q‐S). In addition, we also used high glucose or ‐glucosamine medium to incubate wild‐type (P53^+/+^) HeLa and P53 knock‐out (P53^−/−^) HeLa cells. P53 knockout could suppress EPB41L4A‐AS1 up‐regulation (Supporting information Figure S2F‐G). These data suggest that EPB41L4A‐AS1 up‐regulation induced by persistently high glucose or glucosamine concentrations primarily occurred via TP53 over‐expression.

### 2.3 EPB41L4A‐AS1 regulates glucose uptake and mitochondrial respiration

**FIGURE 3:**
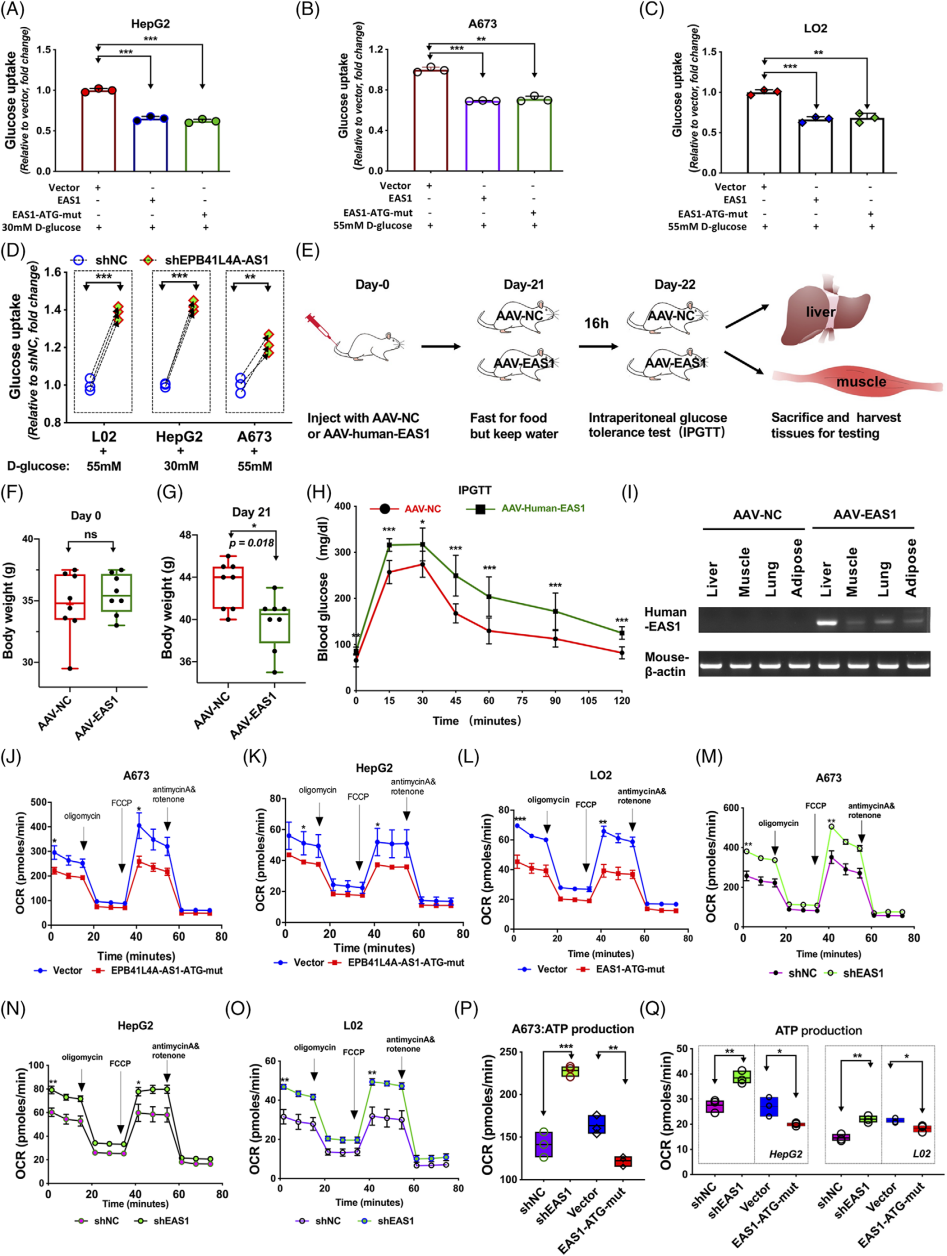
EPB41L4A‐AS1 regulates glucose uptake and mitochondrial respiration. As shown, EAS1 represents EPB41L4A‐AS1; (A‐D) Glucose uptake in cells treated at high glucose concentrations (n = 3). (E) Schematic representation of the animal experiments. (F‐G) Body weight of mice at days 0 and 21. (H) Measurement of blood glucose levels after AAV‐NC mice (n = 8) and AAV‐EAS1 mice (n = 8) were injected with 10% glucose injection (2 mg/g body weight) at 0, 15, 30, 45, 60, 90 and 120 min. (I) RT‐PCR detection of human lncRNA EPB41L4A‐AS1 expression in the liver, muscle, lung and adipose tissues of AAV‐NC and AAV‐EAS1 mice, using mouse β‐actin as the loading control. (J‐L) The oxygen consumption rate (OCR) measured using the Seahorse XFp assay in cells treated at high glucose concentrations for 24 h. (M‐O) The OCR was measured (using the Seahorse XFp assay) in cells treated at high glucose concentrations for 24 h after the stable knockdown of EPB41L4A‐AS1 (n = 3). (P‐Q) ATP production estimated using the Seahorse XFp mitochondrial respiration assay (n = 3)

Previously, we reported that EPB41L4A‐AS1 down‐regulation in tumor cells is involved in the enhancement of glycolysis in tumors.^28^ However, the mechanism underlying the up‐regulation of EPB41L4A‐AS1 in T2DM remains unknown. According to RNA‐seq data from the GTEx database, EPB41L4A‐AS1 expression was found to be relatively lower in normal tissues with a high demand for glucose such as liver, muscle and heart tissues (Supporting information Figure S3A). These findings indicate that EPB41L4A‐AS1 might function as a repressor of glucose uptake. Through gene structure analysis, the EPB41L4A‐AS1 gene was found to generate both the lncRNA EPB41L4A‐AS1 and TIGA1 peptide.^39^ To distinguish between the functions of EPB41L4A‐AS1 and the TIGA1 peptide, we constructed plasmids of EPB41L4A‐AS1 and EPB41L4A‐AS1 with a TIGA1 ATG mutation (named EPB41L4A‐AS1‐ATG‐mut) (these could not express TIGA1 protein because of the ATG mutation) (Supporting information Figure S3B), over‐expressed these plasmids in HepG2, A673 and L02 cells, and incubated the cells under high glucose concentrations for 24 h (Supporting information Figure S3C and D). All cells over‐expressing EPB41L4A‐AS1 or EPB41L4A‐AS1‐ATG‐mut exhibited reduced glucose uptake ability. Furthermore, the glucose uptake remained unchanged in cells over‐expressing TIGA1 peptides compared to that in cells over‐expressing EPB42L4A‐AS1 alone (Figure 3A‐C). Conversely, we also developed stable EPB41L4A‐AS1 knock‐down cell lines (Supporting information Figure S3E) and induced these with high levels of glucose. The glucose uptake was significantly enhanced in these cells (Figure 3D). These results suggest that EPB41L4A‐AS1 functions as a repressor of glucose uptake.

Animal experiments were conducted to study whether EPB41L4A‐AS1 could also regulate glucose homeostasis in vivo. Because of the low conservation of the lncRNA EPB41L4A‐AS1 sequence between humans and mice (Supporting information Figure S3F), we expressed human lncRNA EPB41L4A‐AS1 in mice using the adeno‐associated virus (AAV) system.^40^ To express human lncRNA EPB41L4A‐AS1 in mice, a plasmid expressing human EPB41L4A‐AS1 (AAV‐EAS1) and a control plasmid (AAV‐NC) were packaged into AAV‐DJ, which contained eight different natural serotypes and could efficiently infect various tissues and cells. The mice were injected with AAV‐NC or AAV‐EAS1 at a titre of 1.5 × 10^11^ per mouse. At 21 days after injection, we found that the body weight gain in mice injected with AAV‐EAS1 was lower than that in AAV‐NC mice (Figure 3E‐G). Moreover, the results of the intra‐peritoneal glucose tolerance test (IPGTT) showed that the fasting blood glucose level (at time 0) in mice expressing human lncRNA EPB41L4A‐AS1 (AAV‐EAS1) was higher than that in control mice (AAV‐NC) (Figure 3H). In addition, mice injected with AAV‐EAS1 showed higher blood glucose levels in response to a high glucose load, which was suggestive of impaired glucose tolerance (Figure 3H). In addition, the expression of human lncRNA EPB41L4A‐AS1 was confirmed using RT‐PCR in mice liver, muscle, lung and adipose tissues (Figure 3I). We suggest that, consistent with the function of lncRNA EPB41L4A‐AS1 in human cells, human lncRNA EPB41L4A‐AS1 could act as a cellular glucose uptake repressor in both humans and mice.

Since a decreased metabolic glucose level is a hallmark of T2DM, we performed an oxygen consumption rate (OCR) assay to analyze the effect of EPB41L4A‐AS1 on mitochondrial respiration. We over‐expressed lncRNA EPB41L4A‐AS1 (EPB41L4A‐AS1‐ATG‐mut) in L02, A673 and HepG2 cells, and then incubated the cells in high glucose media. The OCR assay revealed that mitochondrial respiration was reduced in lncRNA EPB41L4A‐AS1‐over‐expressing cells (Figure 3J‐L, Supporting information Figure S4A‐C) but enhanced in cells with stable EPB41L4A‐AS1 knockdown (Figure 3M‐O, Supporting information Figure S4D‐F). ATP production decreased notably when EPB41L4A‐AS1‐ATG‐mut was over‐expressed, but increased when EPB41L4A‐AS1 was knocked down (Figure 3P and Q). In addition, these changes did not result from the changes in cell number during the incubation experiment (Supporting information Figure S4G‐I). These data demonstrate that EPB41L4A‐AS1 negatively regulates glucose uptake and inhibits mitochondrial respiration in T2DM cell models.

### 2.4 EPB41L4A‐AS1 regulates glucose uptake through glucose transporters

**FIGURE 4:**
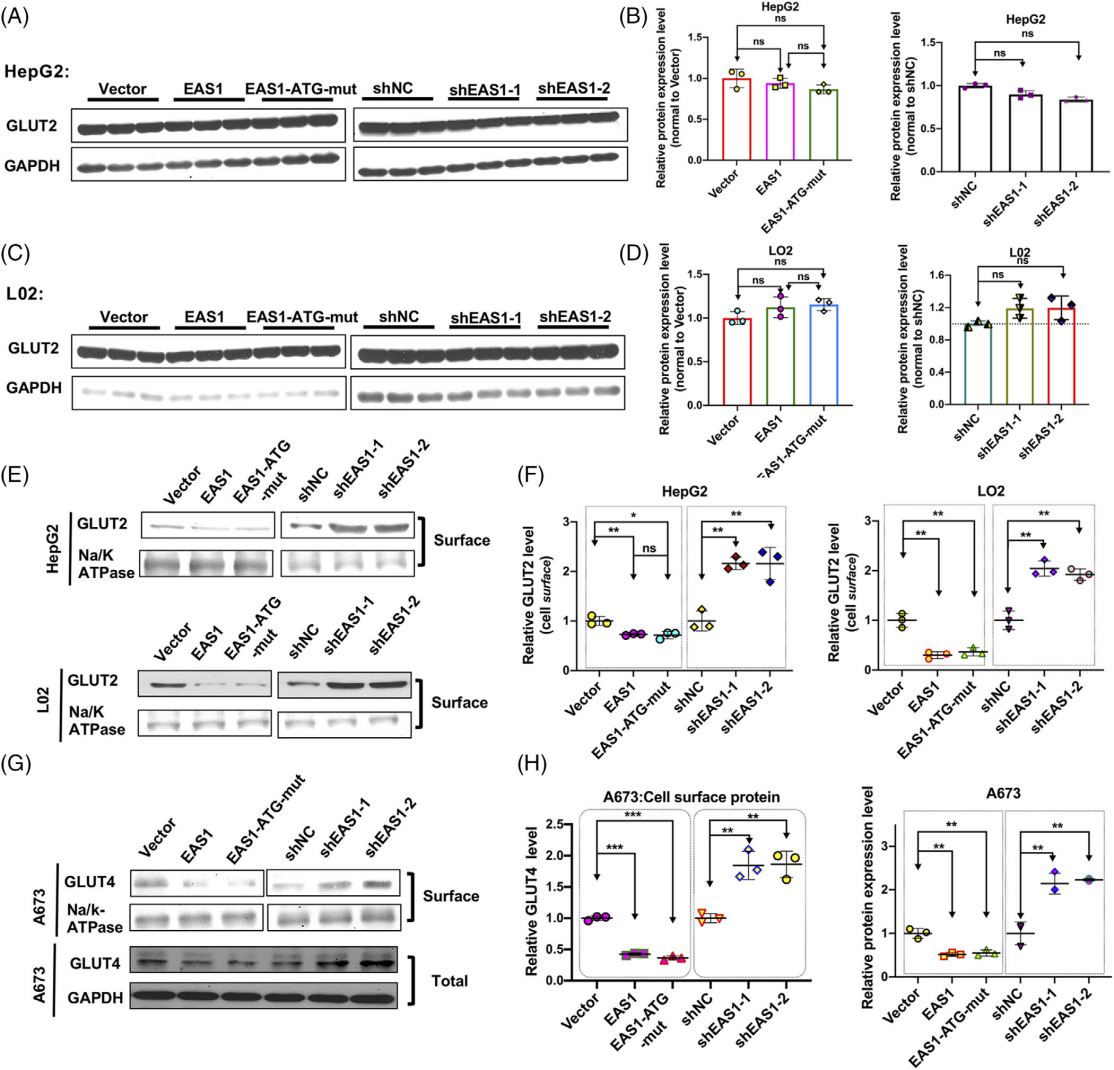
EPB41L4A‐AS1 regulates glucose uptake through GLUT2 or GLUT4. As shown, EAS1 represents EPB41L4A‐AS1; (A‐D) GLUT2 protein level assayed by western blotting of proteins from HepG2 (n = 3) or L02 (n = 3) cells with transient EPB41L4A‐AS1/EPB41L4A‐AS1‐ATG‐mut over‐expression or stable EPB41L4A‐AS1 knockdown, Student's *t*‐test. (E‐F) Cell membrane surface GLUT2 protein levels in HepG2 (n = 3) or L02 (n = 3) cells stimulated with 100 nM insulin for 30 min after the transient over‐expression of EPB41L4A‐AS1/EPB41L4A‐AS1‐ATG‐mut for 48 h or after the stable knockdown of EPB41L4A‐AS1, as measured by using western blotting. (G‐H) Total GLUT4 protein level (n = 3) and cell membrane surface GLUT4 protein level (n = 3) in A673 cells stimulated with 100 nM insulin for 30 min after the transient over‐expression of EPB41L4A‐AS1/EPB41L4A‐AS1‐ATG‐mut for 48 h or after the stable knockdown of EPB41L4A‐AS1, as measured using western blotting

We studied the effect of EPB41L4A‐AS1 on glucose uptake. In accordance with the tissue specificity of glucose transporters, hepatocytes and muscle cells primarily take up glucose via GLUT2/SLC2A2 and GLUT4, respectively. We investigated whether EPB41L4A‐AS1 regulates glucose uptake through GLUT2 or GLUT4. Immunoblotting assays revealed that the GLUT2 protein levels remained unchanged, regardless of whether lncRNA EPB41L4A‐AS1 was over‐expressed or knocked down, in HepG2 or L02 cells (Figure 4A‐D). Because GLUT2 and GLUT4 both accelerate glucose uptake by translocating to the cell surface, we isolated cell‐surface proteins to assess whether EPB41L4A‐AS1 affected the endocytosis of GLUT2. A biotinylated cell‐surface protein‐labelling assay showed that the cell‐surface GLUT2 protein levels were reduced when EPB41L4A‐AS1 was over‐expressed, whereas the stable knockdown of EPB41L4A‐AS1 exerted an opposite effect (Figure 4E and F, Supporting information Figure S5A and B). In addition, TIGA1 over‐expression did not affect GLUT2 endocytosis. In contrast to GLUT2 expression, GLUT4 expression was significantly decreased in lncRNA EPB41L4A‐AS1‐over‐expressing A673 cells, but enhanced in stable EPB41L4A‐AS1 knock‐down A673 cells (Figure 4G and H, Supporting information Figure S5C). Moreover, the expression of GLUT4 on the cell surface was markedly decreased when EPB41L4A‐AS1 or EPB41L4A‐AS1‐ATG‐mut was over‐expressed and markedly increased when EPB41L4A‐AS1 was stably knocked down (Figure 4G and H, Supporting information Figure S5D). These data indicated that the lncRNA EPB41L4A‐AS1, and not the TIGA1 peptide, regulated GLUT2/4 endocytosis and GLUT4 protein levels.

### 2.5 EPB41L4A‐AS1 negatively regulates GLUT4 transcription

**FIGURE 5:**
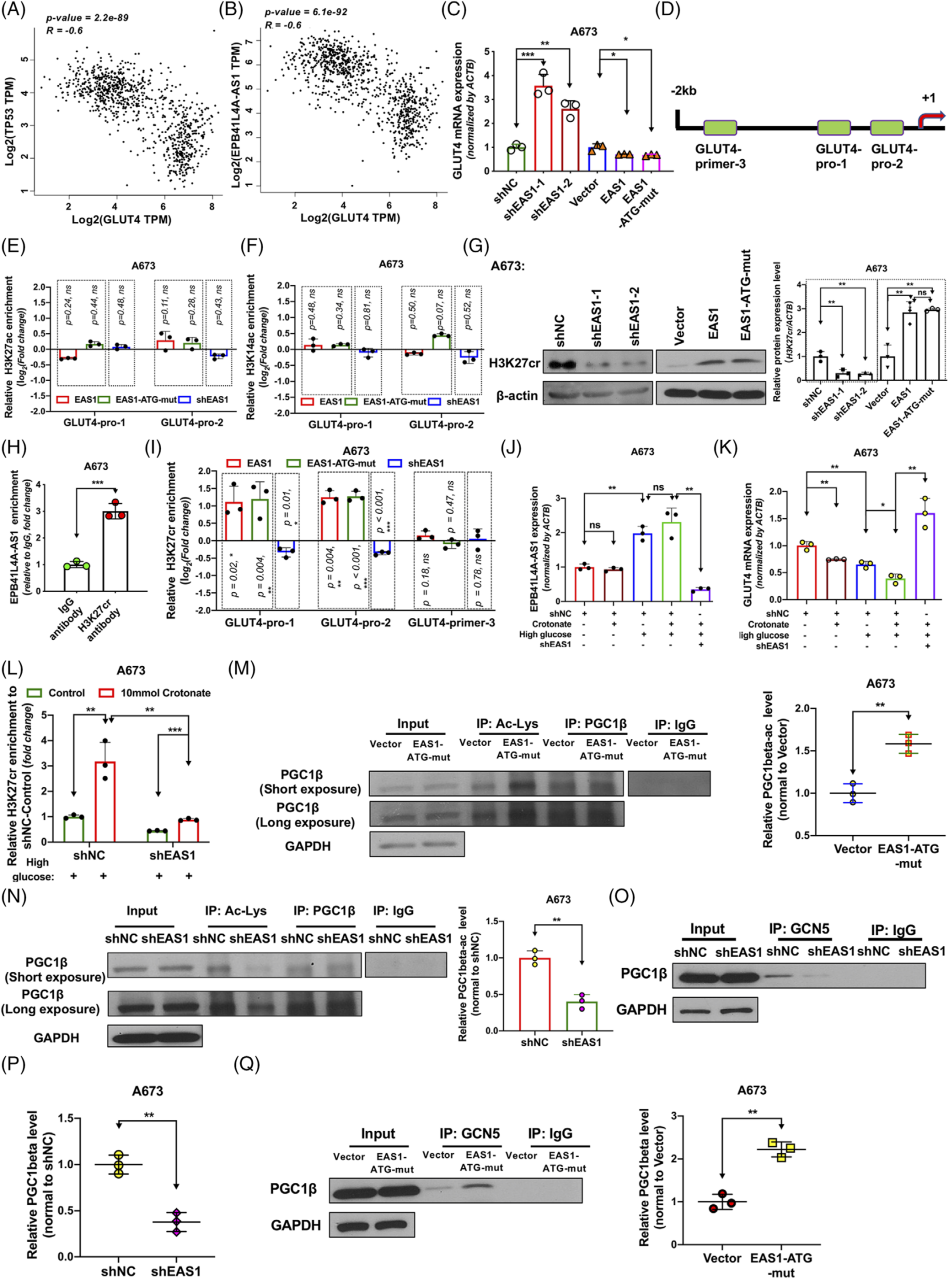
EPB41L4A‐AS1 negatively regulates GLUT4 transcription by increasing H3K27 crotonylation and PGC1‐β lysine acetylation. As shown, EAS1 represents EPB41L4A‐AS1; (A‐B) Pearson's correlation analysis of TP53 or EAS1 and GLUT4 (SLC2A4) expression in human muscle and adipose tissues. Data were obtained from the GTEx RNA‐seq database, and images were obtained from the GEPIA website. (C) GLUT4 mRNA levels measured using qPCR after the over‐expression of EAS1/EAS1‐ATG‐mut for 48 h or after the stable knockdown of EAS1 (n = 3), Student's *t*‐test. (D) Primer locations in the GLUT4 promoter, as designed for chromatin immunoprecipitation‐qPCR. (E) H3K27ac enrichment in the GLUT4 promoter in A673 cells with EAS1/EAS1‐ATG‐mut over‐expression or EAS1 knockdown (n = 3). Data shown as log_2_(fold change); EAS1 and EAS1‐ATG‐mut expression was normalized to that of the vector (control, value = 0), whereas shEAS1 expression was normalized to that of shNC (control, value = 0). (F) H3K14ac enrichment in the GLUT4 promoter in A673 cells after the over‐expression of EAS1 or EAS1‐ATG‐mut plasmids for 48 h and in A673 cells with stable EAS1 knockdown (n = 3). (G) Total H3K27cr levels were measured by immunoblotting using stable EAS1 knock‐down A673 cells or A673 cells with EAS1 or EAS1‐ATG‐mut over‐expression (n = 3). (H) Interaction between EPB41L4A‐AS1 and H3K27cr (n = 3). (I) H3K27cr enrichment in the GLUT4 promoter was measured with the ChIP assay (n = 3). (J‐K) A673 cells were incubated with 10 mM crotonate or at high glucose concentrations or both for 24 h, and qRT‐PCR was conducted to measure the EPB41L4A‐AS1 (J) and GLUT4 levels (K) (n = 3). (L) shNC and shEAS1 A673 cells were incubated with or without glucose at high concentrations and 10 mM crotonate for 24 h, and ChIP‐qPCR was conducted to detect the occupation of H3K27cr in the GLUT4 promoter region (n = 3). (M‐N) Immunoprecipitation for measuring the level of lysine acetylation in PGC1‐β in A673 cells (n = 3). (O‐Q) GCN5 and PGC1‐β interactions were analyzed by the immunoprecipitation assay in stable EAS1 knock‐down A673 cells (n = 3) and EAS1‐ATG‐mut‐over‐expressing A673 cells (n = 3)

To further study how EPB41L4A‐AS1 regulates the GLUT4 protein levels to affect glucose uptake, we analyzed the RNA‐seq data for EPB41L4A‐AS1, TP53 and GLUT4 from the GTEx database. Pearson's correlation analysis showed that GLUT4 expression negatively correlated with the expression of TP53 (*r = *−0.6*, p* value = 2.2e‐89) or EPB41L4A‐AS1 (*r = −*0.6, *p* value = 6.1e‐92) (Figure 5A and B). We also performed a quantitative PCR (qPCR) assay using A673 cells and found that EPB41L4A‐AS1 regulated the GLUT4 mRNA levels (Figure 5C). These results suggest that EPB41L4A‐AS1 regulates GLUT4 transcription. As epigenetic regulation is one of the primary gene regulation mechanisms used by lncRNAs, especially during histone modification, we next studied whether EPB41L4A‐AS1 regulated GLUT4 expression. Chromatin immunoprecipitation (ChIP)‐qPCR assays showed that the enrichment of H3K27ac in the GLUT4 promoter region induced no apparent change in EPB41L4A‐AS1‐over‐expressing or stable EPB41L4A‐AS1 knock‐down A673 cells (Figure 5D and E). The occupation of H3K14ac in the GLUT4 promoter region was also detected, which showed similar results (Figure 5F).

Our group has previously reported that H3K27 crotonylation is negatively associated with gene expression.^21^ In addition, another study showed that an increase in H3K9cr reading by Taf14 reduced gene expression during nutrient limitation.^41^ Based on these findings, we next investigated whether EPB41L4A‐AS1 regulates histone crotonylation. Immunoblotting results showed that the global H3K27cr levels were reduced when EPB41L4A‐AS1 was silenced, whereas the H3K27cr levels were increased when lncRNA EPB41L4A‐AS1 was over‐expressed (Figure 5G and Supporting information Figure S6A). Moreover, we found that EPB41L4A‐AS1 interacted with H3K27cr (Figure 5H). These results indicated that EPB41L4A‐AS1 affects histone crotonylation. To investigate whether EPB41L4A‐AS1 regulates GLUT4 expression via H3K27cr, lncRNA EPB41L4A‐AS1‐over‐expressing or lncRNA EPB41L4A‐AS1‐deleted cells were used to detect H3K27cr occupation in the GLUT4 promoter region. ChIP‐qPCR revealed that the over‐expression of lncRNA EPB41L4A‐AS1 could increase H3K27cr occupation. Additionally, the knockdown of EPB41L4A‐AS1 attenuated H3K27cr enrichment (Figure 5I). The cells were incubated with crotonate, the donor for crotonylation, for 24 h. Exogenous crotonate alone did not alter the EPB41L4A‐AS1 expression levels but decreased GLUT4 mRNA expression in moderation. When treated only with glucose at a high concentration, the EPB41L4A‐AS1 expression levels were markedly increased, and GLUT4 expression was decreased in moderation. However, when the cells were incubated with crotonate under high glucose concentrations, the expression levels of both EPB41L4A‐AS1 and GLUT4 changed considerably. EPB41L4A‐AS1 knockdown abolished the inhibition of GLUT4 expression induced by high glucose and crotonate concentrations (Figure 5J and K). Among the obvious changes associated with EPB41L4A‐AS1 and GLUT4 expression between shNC and shEAS1 A673 cells treated with both crotonate and excess glucose, we detected H3K27cr enrichment at the GLUT4 promoter, indicating that EPB41L4A‐AS1 knockdown markedly decreased the occupation of H3K27cr in the GLUT4 promoter (Figure 5L). These data suggest that EPB41L4A‐AS1 regulates GLUT4 transcription through changes in H3K27cr occupation in the promoter region. Similar to histone acetylation, histone crotonylation can affect transcription factor binding in the promoter region to regulate transcription. PGC1‐β is a transcriptional co‐activator that was reported to promote GLUT4 transcription, and its transcriptional activity was shown to be repressed by lysine acetylation mediated by GCN5/lysine acetyltransferase 2A (KAT2A) at 10 lysine residues: K202, K218, K230, K455, K469, K645, K674, K726, K933 and K994; these sites were distributed across the major domains of PGC1‐β.^42^ We further investigated whether EPB41L4A‐AS1 affected PGC1‐β expression. We found that the PGC1‐β and GCN5 protein levels remained unchanged when EPB41L4A‐AS1 was knocked down (Supporting information Figure S6B and C). Next, a co‐immunoprecipitation assay revealed that the over‐expression of lncRNA EPB41L4A‐AS1 enhanced the total proportion of PGC1‐β acetylation (Figure 5M, Supporting information Figure S6D and E). However, the inhibition of EPB41L4A‐AS1 reduced the proportion of total PGC1‐β acetylation (Figure 5N, Supporting information Figure S6F and G). The binding between GCN5 and PGC1‐β indicated the same trend (Figure 5O‐Q, Supporting information Figure S6H). The above results indicated that the lncRNA EPB41L4A‐AS1 enhanced H3K27cr occupation in the GLUT4 promoter region. The proportion of PGC1‐β acetylation was also enhanced by EPB41L4A‐AS1. The two phenomena occurred simultaneously to inhibit the expression of GLUT4.

### 2.6 EPB41L4A‐AS1 regulates H3K27cr via interaction with GCN5

**FIGURE 6:**
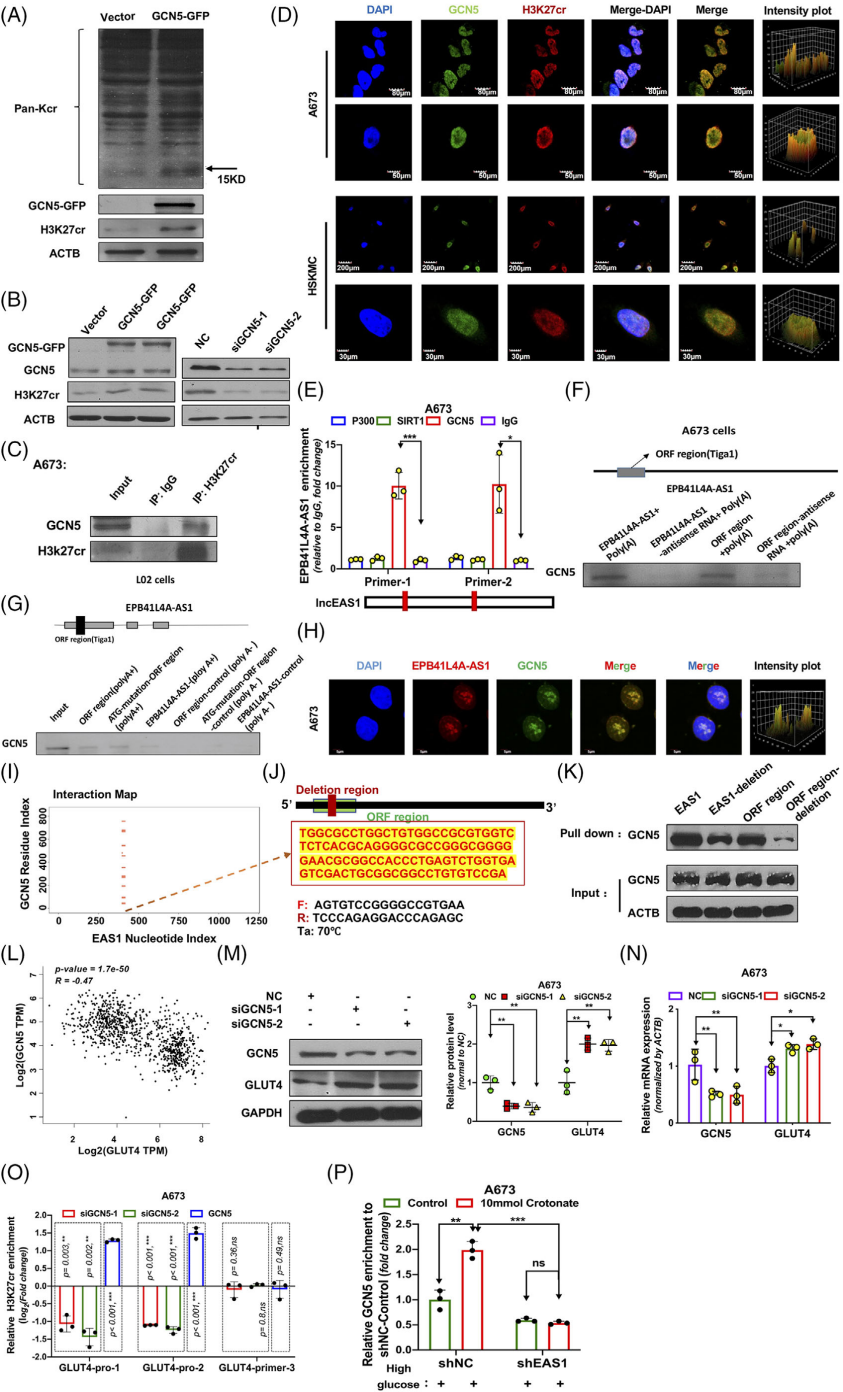
EPB41L4A‐AS1 regulates H3K27cr via interaction with GCN5. As shown, EAS1 represents EPB41L4A‐AS1; (A) Immunoblot‐assayed pan crotonyl lysine of proteins and H3K27cr in HepG2 cells with or without GCN5 over‐expression. (B) The total H3K27cr levels were tested by western blotting after GCN5 knockdown or GCN5‐GFP over‐expression in A673 cells. (C) Immunoprecipitation analysis of the interaction between H3K27cr and GCN5 in A673 cells. (D) H3K27cr and GCN5 co‐localization in A673 cells and human primary skeletal muscle cells (HSkMC) was detected by immunofluorescence. (E) EPB41L4A‐AS1 interaction with GCN5 in A673 cells evaluated by RNA immunoprecipitation‐qPCR analysis (n = 3). Two different primers were designed to detect EPB41L4A‐AS1. (F) Interaction between EPB41L4A‐AS1 RNA and GCN5 in A673 cells evaluated using the RNA pull‐down assay, with each anti‐sense RNA as a control. (G) EPB41L4A‐AS1 RNA and GCN5 interaction in L02 cells evaluated using the RNA pull‐down assay, RNA without poly(A) as the negative control. (H) Co‐localization of EPB41L4A‐AS1 RNA (red) and GCN5 protein (green) in A673 cells. (I) The binding sites between lncRNA EPB41L4A‐AS1 and GCN5 predicted using catRAPID. (J) The diagram shows the deletion region of EPB41L4A‐AS1. (K) EPB41L4A‐AS1 and the ORF region with or without binding sites, predicted using catRAPID deletion, were used for the RNA pull‐down assay, and GCN5 expression was measured by western blotting. (L) Pearson's correlation analysis of GCN5 and GLUT4 expression in human muscle and adipose tissues. Data were obtained from the GTEx RNA‐seq database, and images were obtained from the GEPIA website. (M‐N) GLUT4 protein (n = 3) and mRNA (n = 3) levels were assayed after the transient knockdown of GCN5 in A673 cells (n = 3). (O) H3K27cr enrichment in the GLUT4 promoter in A673 cells with or without GCN5 knockdown or GCN5 over‐expression (n = 3), as measured by chromatin immunoprecipitation‐qPCR. Data shown as log_2_(fold change); siGNC5‐1 and siGCN5‐2 expression was normalized to that of the negative control (NC) (control, value = 0), whereas GCN5 expression was normalized to that of the vector (control, value = 0), Student's *t*‐test. (P) GCN5 enrichment in the GLUT4 promoter region was measured after A673 cells were incubated with 10 mM crotonate at high glucose concentrations for 24 h (n = 3)

We next studied the molecular mechanism by which EPB41L4A‐AS1 regulates H3K27cr. Because many studies have demonstrated that crotonylation and acetylation share the same enzyme system, we focused on acetyl transferases. Our previous study reported that EPB41L4A‐AS1 does not interact with EP300,^28^ the writer for both the crotonylation and acetylation of histones. Gcn5, another lysine acetylation writer, was reported to regulate histone crotonylation in budding yeast.^43^ Therefore, we next determined whether GCN5 could regulate the crotonylation of histone 3 at the K27 site in human cells. First, we over‐expressed GCN5 in A673 cells and found that the total H3K27cr levels increased, whereas GCN5 knockdown reduced the total H3K27cr levels (Figure 6A and B). In addition, co‐immunoprecipitation revealed that H3K27cr interacted with GCN5 (Figure 6C and Supporting information Figure S7A), which was also supported by findings from the immunofluorescence assay (Figure 6D). These results suggest that GCN5 regulates histone crotonylation, especially at lysine residues at the H3K27 site.

As lncRNAs usually regulate the epigenetic modification of lysine by interacting with and recruiting related functional proteins to target proteins, we assumed that EPB41L4A‐AS1 could interact with GCN5. First, the RNA‐protein interaction prediction (RPISeq) bioinformatics tool was used to analyze the possibility of EPB41L4A‐AS1 and GCN5 interaction^44^ to confirm this hypothesis. The results indicated that the two molecules could interact with each other (RF = 0.85, SVM = 0.98). For in vitro experimental verification, RNA immunoprecipitation (RIP)‐qPCR showed that GCN5 protein could pull‐down EPB41L4A‐AS1 (Figure 6E). Conversely, an RNA pull‐down assay was also conducted using the A673 nuclear lysate, and lncRNA EPB41L4A‐AS1 was found to interact with GCN5 (Figure 6F). The same result was obtained with RNA pull‐down in L02 cell lysates (Figure 6G). Moreover, RNA‐fluorescent in‐situ hybridization (RNA‐FISH) followed by immunofluorescence estimation showed that EPB41L4A‐AS1 co‐localized with GCN5 (Figure 6H). To further study the interaction between EPB41L4A‐AS1 and GCN5, we used the catRAPID database to predict the potential binding sites; the results indicated that the binding sites were just contained in the ORF region (Figure 6I and J). Consistent with this prediction, we confirmed the binding between the ORF region and GCN5 in RNA pull‐down assays (Figure 6F and G). For further validation, we constructed a plasmid containing EPB41L4A‐AS1 with the predicted binding‐region deletion (EAS1‐deletion) and a plasmid containing the ORF region with the same predicted binding‐region deletion (ORF region‐deletion). Next, an RNA pull‐down assay was performed, and the results showed that the pull‐down of GCN5 decreased significantly after the predicted deletion of the binding region of EPB41L4A‐AS1. Of note, binding to GCN5 nearly could not be completed when the ORF region with the predicted binding region was deleted (Figure 6K). These data demonstrate that EPB41L4A‐AS1 is interacting with GCN5. Furthermore, we also analyzed the correlation of GCN5 and GLUT4 RNA expression in human normal muscle and adipose tissues; the results indicated a negative correlation (Figure 6L). GCN5 knockdown enhanced GLUT4 expression (Figure 6M and N, Supporting information Figure S7B). Furthermore, to investigate the effect of GCN5 on GLUT4 expression, we measured H3K27cr occupation in the GLUT4 promoter region. We found that GCN5 inhibition repressed the enrichment of H3K27cr in the GLUT4 promoter region, whereas the over‐expression of GCN5 exerted the opposite effect (Figure 6O). When A673 cells with stable EPB41L4A‐AS1 knockdown were incubated with 10 mM crotonate, the donor for lysine crotonylation, under high glucose levels for 24 h, we found no noticeable change in GCN5 enrichment at GLUT4 promoter region compared to that in cells without stable EPB41L4A‐AS1 knockdown (Figure 6P). This indicated that EPB41L4A‐AS1 was essential for the GCN5‐mediated regulation of H3K27cr enrichment in the GLUT4 promoter, but the EPB41L4A‐AS1/GCN5 complex was not the sole regulator of H3K27cr levels in the GLUT4 promoter region (Figures 5L and 6P).

### 2.7 EPB41L4A‐AS1 regulates GLUT4/2 endocytosis by TXNIP

**FIGURE 7:**
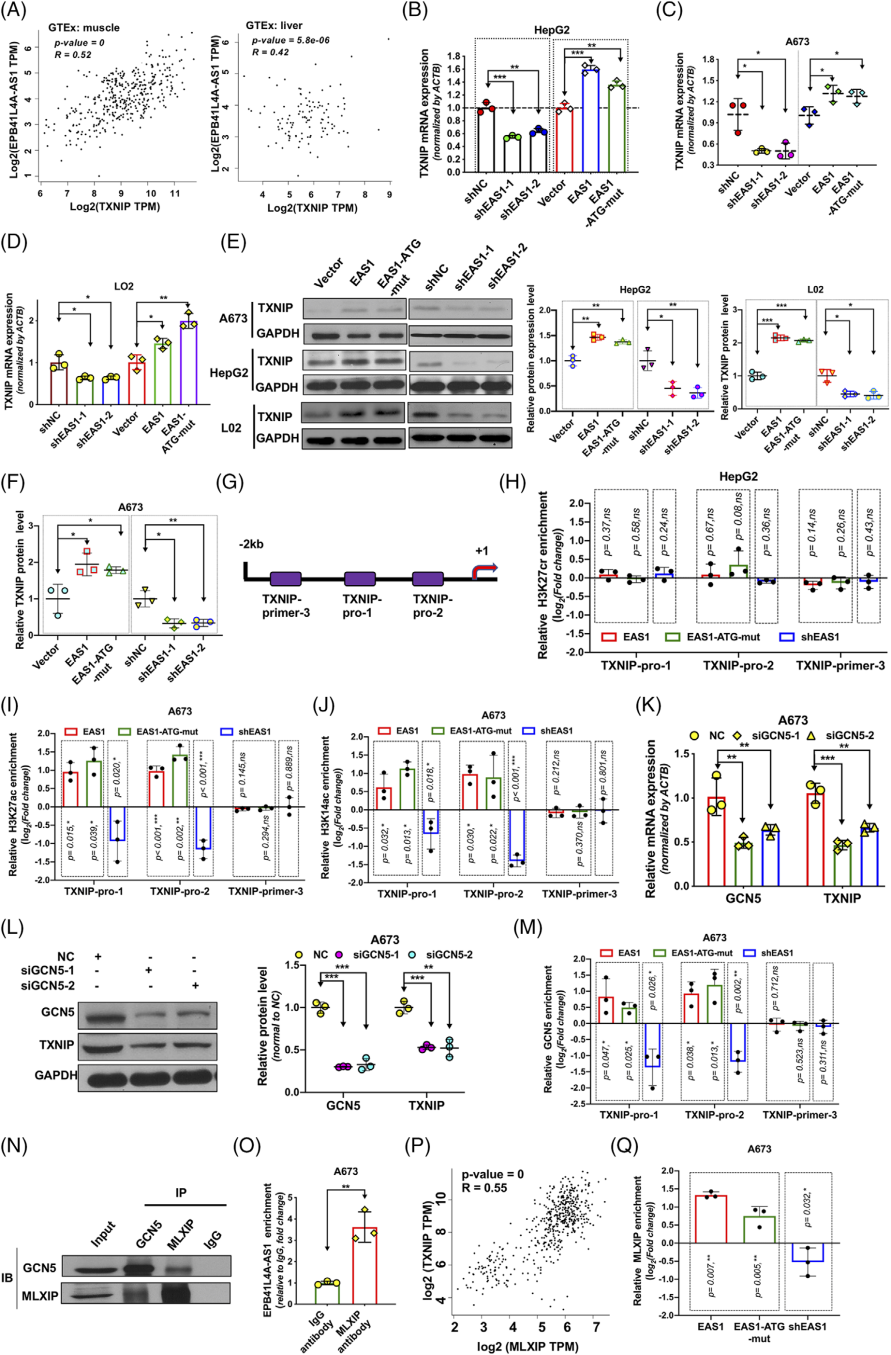
*EPB*41L4A‐AS1 regulates TXNIP transcription via enhancement of H3K14 and H3K27 acetylation. As shown, EAS1 represents EPB41L4A‐AS1; (A) TXNIP and EPB41L4A‐AS1 expression correlation in muscle and liver tissues; data obtained from GTEx database and the GEPIA website are plotted in the diagram. (B‐D) The mRNA expression of TXNIP (n = 3) measured using qRT‐PCR. (E‐F) TXNIP protein levels in HepG2 (n = 3), A673 (n = 3),and L02 (n = 3) cells after the stable knockdown of EPB41L4A‐AS1 or the transient over‐expression of EPB41L4A‐AS1 or EPB41L4A‐AS1‐ATG‐mut, as measured using western blotting. (G) Schematic representation of the primer location in the TXNIP promoter region. (H) H3K27cr enrichment in the TXNIP promoter measured by chromatin immunoprecipitation (ChIP)‐qPCR in HepG2 cells over‐expressing EPB41L4A‐AS1 or EPB41L4A‐AS1‐ATG‐mut or with EPB41L4A‐AS1 knockdown (n = 3), Student's *t*‐test. (I‐J) H3K27ac and H3K14ac enrichment in the TXNIP promoter of A673 cells after the over‐expression of EPB41L4A‐AS1 or EPB41L4A‐AS1‐ATG‐mut or the stable knockdown of EPB41L4A‐AS1 (n = 3), as evaluated using ChIP‐qPCR analysis, Student's *t*‐test. (K‐L) GCN5 was transiently knocked down in A673 cells for 48 h using GCN5‐specific siRNAs, and the GCN5 and TXNIP protein and mRNA levels were measured by immunoblotting (n = 3) and qPCR (n = 3), respectively. (M) GCN5 occupation in the TXNIP promoter in A673 cells (n = 3) was assayed by ChIP‐qPCR. (N) The interaction between MLXIP and GCN5 in A673 cells. (O) RNA immunoprecipitation‐qPCR analysis of the interaction between EPB41L4A‐AS1 and MLXIP in A673 cells (n = 3). (P) MLXIP and TXNIP RNA expression correlation determined by Pearson's correlation analysis in muscle and liver tissues. Data were obtained from the GTEx database, and the diagram was obtained from the GEPIA website. (Q) MLXIP enrichment at the TXNIP promoter in A673 cells after the stable knockdown of EPB41L4A‐AS1 or over‐expression of EPB41L4A‐AS1 or EPB41L4A‐AS1‐ATG‐mut (n = 3), as detected using ChIP‐qPCR

In the above sub‐sections, we have stated that EPB41L4A‐AS1 can regulate GLUT2/4 endocytosis to further affect glucose uptake (Figure 4E‐H). To clarify this process, we conducted a thorough investigation of the underlying molecular mechanisms. TXNIP has been reported to function as a negative regulator of glucose uptake^45^, ^46^ that regulates glucose uptake primarily as an adaptor for the endocytosis of glucose transporters such as Glut1–4.^47^, ^48^ Thus, we investigated whether EPB41L4A‐AS1 affected glucose uptake via the regulation of TXNIP. Correlation analysis revealed a positive correlation between TXNIP and EPB41L4A‐AS1 RNA expression in human normal liver (*r* = 0.42*, p *= 5.8e‐06) and muscle (*r *= 0.5*2, p *= 0) tissues (Figure 7A). The over‐expression of lncRNA EPB41L4A‐AS1 enhanced TXNIP expression (Figure 7B‐F, Supporting information Figure S8A‐C). Surprisingly, unlike what was observed for GLUT4, the H3K27cr occupation status remained unchanged in the TXNIP promoter region regardless of lncRNA EPB41L4A‐AS1 over‐expression or knockdown (Figure 7G and H). ChIP‐seq data from the ENCODE database showed that H3K27ac was highly abundant around the regulatory region of TXNIP (Supporting information Figure S8D). Therefore, we evaluated H3K27ac occupation, and the results revealed that the H3K27ac levels in the promoter region of TXNIP were markedly increased in exogenous lncRNA EPB41L4A‐AS1‐over‐expressing cells, whereas the opposite effect was observed in cells with EPB41L4A‐AS1 knockdown (Figure 7I and Supporting information S8E and F). Additionally, we also observed a change in the H3K14ac levels in the TXNIP promoter region and found that the changes induced by lncRNA EPB41L4A‐AS1 were similar to those induced by H3K27ac (Figure 7J, Supporting information Figure S8G and H). In short, EPB41L4A‐AS1 regulated TXNIP expression by changing the status of H3K27ac and H3K14ac occupation, but not that of H3K27cr occupation.

To investigate whether the EPB41L4A‐AS1‐binding protein GCN5 mediated this regulation, we first transfected GCN5‐specific siRNAs into cells and measured TXNIP expression, which was found to be reduced (Figure 7K and L, Supporting information Figure S8I). Furthermore, we found that EPB41L4A‐AS1 over‐expression up‐regulated GCN5 binding to the TXNIP promoter, and that the inhibition of EPB41L4A‐AS1 exerted the opposite effect (Figure 7M). These results demonstrate that EPB41L4A‐AS1 positively regulates TXNIP expression by altering H3K27 and H3K14 acetylation via GCN5 in the TXNIP promoter region. Both H3K27ac and H3K14ac could regulate transcription factor binding to gene promoter. MLXIP was reported to bind to the promoter region and promote the transcription of TXNIP, which was shown to interact with GCN5 in the immunoprecipitation assay (Figure 7N). Moreover, the MLXIP protein could also pull down EPB41L4A‐AS1 (Figure 7O). Pearson's correlation analysis also revealed a positive correlation between MLXIP and TXNIP expression in normal liver and muscle tissues (*r *= 0.55, *p* = 0, Figure 7P).^38^ Therefore, we assumed that EPB41L4A‐AS1 regulates TXNIP expression by recruiting the GCN5/MLXIP complex. This assumption was further verified by changes in MXLIP enrichment mediated by EPB41L4A‐AS1 (Figure 7Q).

## 3 DISCUSSION

The lncRNA EPB41L4A‐AS1 was un‐identified until we reported its critical role in the reprogramming of cancer cell metabolism in a previous study.^28^ However, the effects of EPB41L4A‐AS1 on diabetes have not yet been reported. Using rapidly evolving gene‐sequencing technologies, several lncRNAs that regulate diabetes in humans have been identified. EPB41L4A‐AS1 was found to be up‐regulated in patients with T2DM and insulin resistance. The activation of TP53 is an important factor that leads to T2DM development. However, the mechanism by which TP53 contributes to T2DM development remains unclear. In this study, we generated T2DM cell models treated with excess glucose or glucosamine and found that TP53 expression increased in these cell models. Interestingly, the increase in TP53 expression induced by high glucose levels also led to the up‐regulation of EPB41L4A‐AS1 expression. However, the up‐regulation of this lncRNA repressed glucose uptake and mitochondrial respiration via the regulation of GLUT expression or endocytosis, which may further promote hyperglycaemia in patients and accelerate disease development. The knockdown of TP53 nearly blocked the increase in EPB41L4A‐AS1 expression in T2DM cell models induced by excess glucose or glucosamine. In addition, using the AAV system to express human EPB41L4A‐AS1 in mice, we also found that the lncRNA could affect glucose homeostasis in vivo including blood glucose levels and glucose tolerance. These results suggest that EPB41L4A‐AS1 may be a potential therapeutic target for T2DM. However, evidence from additional experiments, such as validation of the findings in primary cell cultures and diabetic tissues, is needed to further support this conclusion.

GLUTs are important for regulating glucose uptake and exhibit pronounced tissue specificity. Muscle cells primarily take in glucose via GLUT4, and liver cells use GLUT2 to exert specific effects on glucose homeostasis regulation. The total GLUT2/GLUT4 protein content and localization of the proteins on the cell membrane surface are the primary limiting factors for glucose uptake. Reportedly, GLUT4 transports glucose by translocating to the cell surface, primarily in muscle and adipose tissues.^49^ We found that the up‐regulation of EPB41L4A‐AS1 affected GLUT4 endocytosis by increasing TXNIP expression. TXNIP acts as a glucose uptake repressor and an adaptor for GLUT4 endocytosis. The stimulation of growth factors, such as insulin, can force TXNIP to dissociate from GLUT4 and inhibit GLUT4 endocytosis, which eventually enhances glucose uptake.

Moreover, because of the high homology among GLUTs, it has been confirmed that TXNIP can regulate the endocytosis of GLUT1, GLUT2 and GLUT3; however, the specific mechanisms underlying GLUT2 and GLUT3 regulation remain unknown.^47^, ^48^ In liver cells, GLUT2, the key glucose uptake transporter, is insulin‐responsive and forms a receptor‐transporter complex with the insulin receptor in the hepatocyte cytomembrane.^50^ We also found that EPB41L4A‐AS1‐mediated TXNIP up‐regulation increased GLUT2 endocytosis and inhibited glucose uptake in liver cells. In addition, EPB41L4A‐AS1 did not alter the total GLUT2 content in liver cells. However, unlike that in liver cells, EPB41L4A‐AS1 up‐regulation was found to regulate the transcriptional activity of PGC1‐β, a transcriptional co‐activator of GLUT4, by affecting the proportion of total PGC1‐β acetylation, which eventually inhibited GLUT4 expression.

LncRNAs usually regulate gene expression through epigenetic mechanisms.^51^, ^52^ Among these, interaction with the histone modification complex seems to be a common mechanism. With respect to the mechanism by which EPB41L4A‐AS1 regulates TXNIP, we first found that EPB41L4A‐AS1 interacts with GCN5, a lysine acetyltransferase, and recruits it to the TXNIP promoter region, thereby, promoting H3K27ac and H3K14ac enrichment. It was previously confirmed that under high glucose concentrations, the transcription level of TXNIP is up‐regulated via MLX/MLXIP, a transcriptional activator of TXNIP.^53^ Here, we further observed that EPB41L4A‐AS1 could bind with and recruit MLXIP to enhance TXNIP expression.

Many studies have reported that histone acetylation and crotonylation are both markers for gene transcription regulation, and both involve a highly consistent enzyme system. For instance, histone acetyltransferase P300 exhibits both acetyltransferase and crotonyltransferase activities, catalysing histone H3K18 crotonylation..^54^ In addition to P300, the histone acetyltransferase MOF also possesses histone crotonyltransferase activities specific for H3K4, H3K9, H3K18 and H3K23, with its activity conserved evolutionarily.^55^ In addition, histone deacetylases, such as HDACs and SIRTs, also exhibit decrotonylase activity in vitro. However, SIRT1/2/3 knockdown did not affect the cellular H3K27cr levels.^56^, ^57^, ^58^ These results indicate that in vivo H3K27 crotonylation is associated with a more complex regulatory mechanism than in vitro crotonylation. GCN5 reportedly regulates histone acetylation and succinylation in human cells and histone crotonylation in yeast, thereby exhibiting the potential to regulate multiple histone modifications. In this study, we found that GCN5 regulates H3K27cr in human cells. GCN5 co‐localized with H3K27cr, and the knockdown of GCN5 reduced the H3K27cr levels, whereas the over‐expression of GCN5 enhanced the H3K27cr levels. Furthermore, EPB41L4A‐AS1 knockdown did not affect GCN5 expression; however, the H3K27cr levels were altered, indicating that EPB41L4A‐AS1 plays a role in regulating histone crotonylation by GCN5. However, additional studies are needed to further investigate the function of GCN5, which regulates histone crotonylation in vivo.

With respect to the role of EPB41L4A‐AS1 in regulating GLUT4 expression, an increase in EPB41L4A‐AS1 expression up‐regulated both GCN5 and H3K27cr localization to the promoter region, which reduced GLUT4 expression. Of note, the inhibition of EPB41L4A‐AS1 reduced the crotonate‐induced increase in H3K27cr occupation in the GLUT4 promoter region, but almost blocked the change in GCN5 enrichment, which suggested that EPB41L4A‐AS1 regulates H3K27cr enrichment by recruiting GCN5 to the GLUT4 promoter region as well as that the EPB41L4A‐AS1/GCN5 complex is an important factor, but not the sole factor, in the regulation of H3K27cr in the GLUT4 promoter region.

**FIGURE 8:**
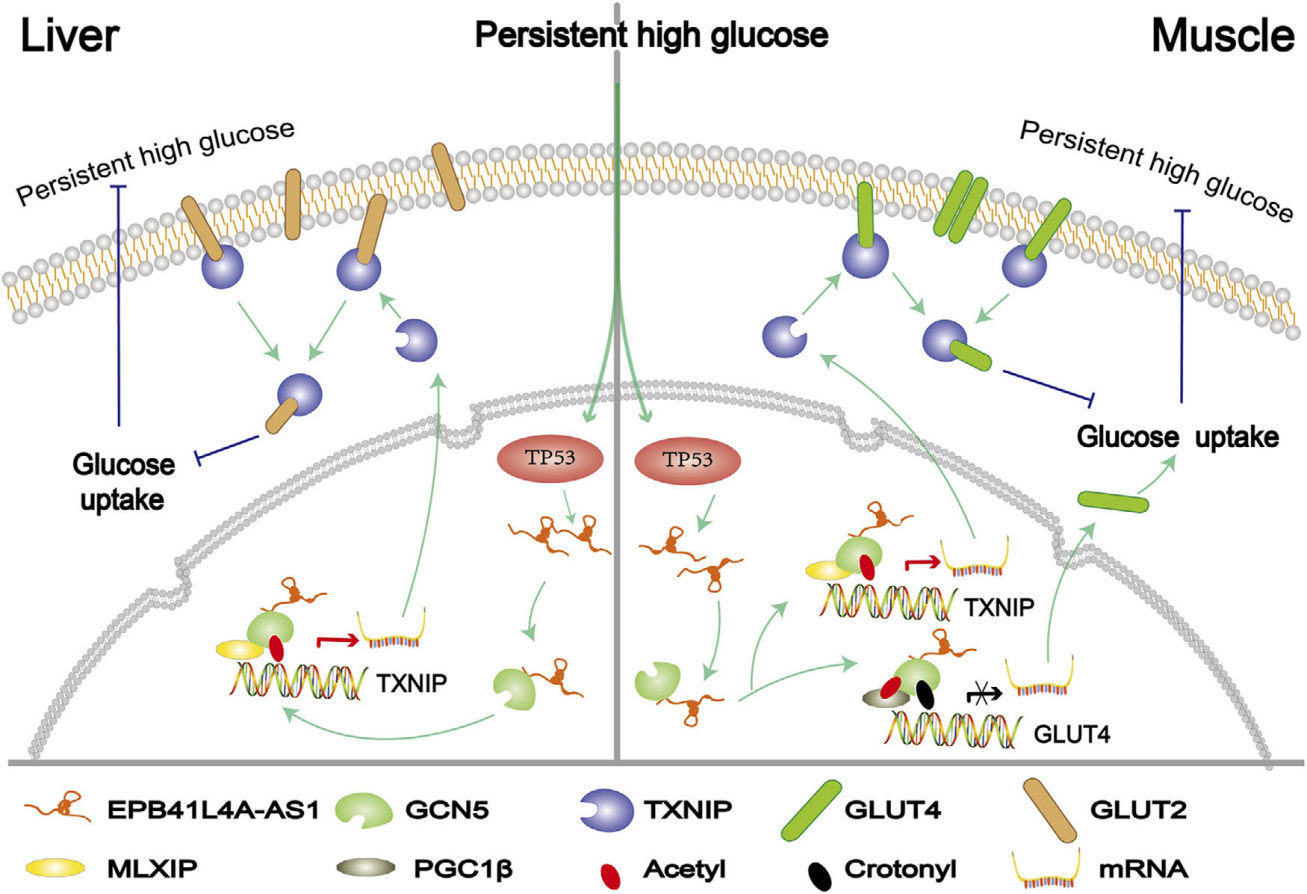
Summary of the molecular mechanism by which EPB41L4A‐AS1 affects glucose uptake. Persistently high glucose concentrations increase the expression of the lncRNA EPB41L4A‐AS1 via the enhancement of TP53 expression. Through interaction with GCN5 and up‐regulation of its own expression, the lncRNA EPB41L4A‐AS1 increases histone H3K27 crotonylation in the GLUT4 promoter region and non‐histone PGC1‐β acetylation, which inhibits GLUT4 transcription and suppresses glucose uptake in muscle cells. Conversely, EPB41L4A‐AS1 binding to GCN5 enhances H3K27 and H3K14 acetylation in the TXNIP promoter region to activate transcription by recruiting the transcriptional activator MLXIP, which enhances GLUT4/2 endocytosis and further suppresses glucose uptake

In summary, we studied the function of EPB41L4A‐AS1 in T2DM cell models, and this is the first study to show that the EPB41L4A‐AS1/GCN5 complex repressed glucose uptake by targeting GLUT4/2 and TXNIP and by regulating histone and non‐histone acetylation or crotonylation (Figure 8). Since a weaker glucose uptake ability is one of the major clinical features of T2DM, the inhibition of EPB41L4A‐AS1 expression seems to be a potentially effective strategy for drug development in T2DM treatment.

## 4 MATERIALS AND METHODS

### 4.1 Cell lines and treatments

The Stem Cell Bank, Chinese Academy of Sciences, kindly provided the HepG2, L02 and A673 cells. Chenxue Biotech provided HSkMC. EdiGene provided HeLa and P53 knock‐out HeLa cells. HepG2 cells were cultured in DMEM with 1 g/L glucose (Gibco, 11885), and HSkMC and A673, L02, HeLa and P53 knock‐out HeLa cells were cultured in DMEM with 4.5 g/L d‐glucose (Gibco, 11965092), with the media supplemented with 10% fetal bovine serum (Biowest). The above‐mentioned cells were maintained in a cell incubator with 5% CO_2_ at 37°C and passaged in dishes with a diameter of 100 mm. Subsequently, HepG2 cells were treated with 30 mM d‐glucose,^33^, ^59^ and HSkMC and L02, A673, HeLa and P53 knock‐out HeLa cells were treated with 55 mM d‐glucose, for 24 h to develop T2DM cell models under a high glucose concentration. All cells were cultured in DMEM supplemented with 5 mM glucosamine for 24 h to develop a T2DM cell model using glucosamine (Sigma‐Aldrich, G1514). The cells were transfected with a lentivirus containing the plasmid shEPB41L4A‐AS1 (GenePharma, Shanghai) and selected using puromycin (Gibco, A11138–03) to obtain stable EPB41L4A‐AS1 knock‐down cell lines. The target sequences of EPB41L4A‐AS1 were as follows: shNC, 5′‐TTCTCCGAACGTGTCACGT‐3′; shEPB41L4A‐AS1‐1, 5′‐GGATGTCCTTGGTGA GGATTT‐3′; shEPB41L4A‐AS1‐2, 5′‐GAGGAGTACT GACATAATTAC‐3′.

### 4.2 Clinical data analysis

Clinical expression data of lncRNA EPB41L4A‐AS1 in GSE23343 and GSE6798 datasets were obtained using R (version 3.5.3 for mac) from GEO database and statistical analysis were carried out using Mann–Whitney U test.

### 4.3 Relative qPCR

Total RNA was harvested and extracted using TRIzol (Takara, D9108B), precipitated with isopropanol, washed with 75% ethanol, and reverse‐transcribed using the TOYOBO FSQ‐201 kit. Relative qPCR was performed using SYBR (TOYOBO, QPK‐201). All experiments were performed in triplicates. The primers (5′‐3′) used for qPCR are shown below: ACTB, F: TGACGTGGACATCCGCAAAG and R: CTGGA AGGTGGACAGCGAGG; EPB41L4A‐AS1 F: CCACCCTGAGTCTGGTGAGT and R: CCTGGCATAGTCGATGATGTA; TP53, F: CCACCATCCACTACAACTACAT and R: AGGACAGGCACAAACACG; GLUT4 F: 5′‐ CCCATTCCTTGGTTCATCGT and R: CCATAGCCTCCGCAACATAC‐3′; TXNIP, F: 5′‐AAAGGATTCTGTGAAG GTGATG and R: TGATTGCCTCTGACTGATGAC‐3′; TXNIP promoter‐1, F: TTAGTGTAACCAGCGGCGTAT and R: CTCCAAATCGAGGAAACCC‐3′; TXNIP promoter‐2, F: CAGGTAGGAAGTGGGAGATA‐3′ and R: CTGTGGAACGGAAAT AGGAG‐3′; TXNIP primer‐3, F: CTAGGCTGTCTGAGGAAGGAA‐3′ and R: CAA GATTGAGATGGGGAGGTA‐3′; GLUT4 promoter‐1, F: ACTCTTTAAGGCGTCAT CTCC and R: AGCAATGCCCCAAAGTAAC‐3′; GLUT4 promoter‐2, F: GGTTGTG ACCACTTGTCCCTC and R: GCTTGGTCCTGTCTTTCAGC‐3′; GLUT4 primer‐3, F: CTACAACCTCCACCTCCT and R: GGCTCATGCCTGTAA TCC ‐3′.

### 4.4 Protein detection

Proteins were isolated using a lysis buffer containing 50 mM Tris‐HCl, 1% Triton X‐100 and 4 mol urea, along with a protease inhibitor cocktail (Roche, 04693132001). The supernatant was used for western blotting analysis. Briefly, protein samples were separated by SDS‐PAGE and transferred to nitrocellulose (NC) membrane filters. Then, 5% milk/TBST was used to block the NC membrane for 1 h. Following this, the primary antibodies were used to treat the NC membrane at 4°C overnight and then for 1.5 h with secondary antibody. The primary antibodies used are as follow: GAPDH (Proteintech, 10494‐1‐AP), TIGA1 (Proteintech, 24698‐1‐AP), TP53 (Proteintech, 10442‐1‐AP), TXNIP (CST,#14715S), GLUT4 (Abcam, ab48547), GLUT2 (Abcam, ab192599), Na/K ATPase (Abcam, ab58475), GCN5/KAT2A (Abcam, ab208097 or ab217876), Pan‐Kcr (PTM Biolab, PTM502), H3K27cr (PTM Biolab, PTM‐526), H3K27ac (Abcam, ab177178), H3K14ac (CST, 7627S), IgG (Abcam, ab171870), MLXIP (Abcam, ab176688), β‐actin (Proteintech, 66009‐1‐lg), PGC1‐β (Abcam, ab176328), acetylated lysine (CST, #9441L) and GFP (Abcam, ab290).

### 4.5 Measurement of glucose levels

Glucose was detected using a glucose test kit (glucose oxidase method) (BIOSINO, China). Briefly, 2 μL of the cell culture medium was mixed with the testing mix and incubated at 37°C for half an hour. The absorbance was measured at 505 nm, and the concentration of glucose in the sample was calculated using standards.

### 4.6 Cell counting kit‐8 assay

The cell number was determined using the cell counting kit—8 (CCK ‐8) (MCE, K0301). Briefly, 6 × 10^3^ cells were inoculated in 96‐well cell culture plates. After being subjected to the different treatments, the culture medium was replaced with 110 μL of the reaction buffer (containing 10 μL of the CCK‐8 buffer), and the cells were incubated for 2.5 h. A microplate reader was carried out for the measurement of absorbance at 450 nm.

### 4.7 Glucose uptake assay

A fluorescence‐based glucose assay kit (BioVision, k666) was used to evaluate the glucose uptake, and 100 nM insulin was used to activate the glucose transporters for 30 min.

### 4.8 Mice

Four‐week‐old wild‐type NIH male mice were generously provided by the Guangdong Medical Laboratory Animal Center (No. 44007200095713) and maintained in an SPF mouse facility for 2 weeks. The mice were divided into two groups to ensure that there was no significant difference in body weight; each group contained eight mice. To express human lncRNA EPB41L4A‐AS1 in mice, plasmids expressing human EPB41L4A‐AS1 (AAV‐EAS1) and a control plasmid (AAV‐NC) were packaged into AAV‐DJ, which contained eight different natural serotypes and could efficiently infect various tissues and cells (GenePharma, Shanghai). AAV‐NC or AAV‐EAS1 were injected at a titre of 1.5 × 10^11^ GC/mL for one mouse with the total volume of 100 μL. At 21 days after injection, the body weights of the mice were recorded, and the mice were fasted for food but provided access to water for 16 h. The IPGTT was performed by administering glucose at 2 mg/g body weight, and the blood glucose levels were measured at 0, 15, 30, 45, 60, 90 and 120 min using a CONTOUR PLUS blood glucose meter (Model 7600P, Bayer). Finally, the mice were sacrificed, and tissues were harvested and total RNA was extracted by TRIzol for detecting human EPB41L4A‐AS1 expression using PCR. The animal experiments were approved by the Animal Research Committee of the Shenzhen International Graduate School of Tsinghua University.

### 4.9 Evaluation of mitochondrial respiration

An Agilent Seahorse XFp Analyzer was warmed overnight. Cells were treated for different purposes before being plated in the cell culture dishes provided with the Seahorse assay (Agilent Technologies 103025–100) and incubated overnight. The probe dish (Agilent Technologies 103059–000) was hydrated overnight in the absence of CO_2_. The cells were incubated in an OCR buffer for 45 min and tested using the Agilent Technologies 103010–100 kit.

### 4.10 ChIP

The cells were cross‐linked with 1% formaldehyde for 18 min, and glycine was used to terminate the cross‐linking. The cells were lysed using a ChIP cell lysis buffer and sonicated (30% power, 5 s, pause for 25 s, 18 cycles). The supernatants were incubated with a ChIP‐grade primary antibody for 2.5 h, following which Dynabeads Protein G was added, and the reaction mixture was rotated at 4°C for another 2 h. The immunoprecipitants were incubated with proteinase K at 50°C for 35 min. Finally, DNA was extracted for qPCR analysis. The antibodies used were as follows: H3K27cr (PTM Biolab, PTM‐526), H3K27ac (Abcam, ab177178), H3K14ac (CST, 7627S), IgG (Abcam, ab171870), MLXIP (Abcam, ab176688) and GCN5/KAT2A (Abcam, ab217876).

### 4.11 Biotinylated cell‐surface protein labelling

Cells were cultured in 100 mm culture dishes, washed with HBSS, labelled with biotin (Thermo Fisher, 21331) on ice for half an hour. Next, the cells were lysed by NP40 buffer for 30 min. After centrifugation (16 000 × *g*, 15 min and 4°C), the supernatants were collected and incubated overnight with 40 μL of the NeutraAvidin Agarose Slurry (Thermo‐Fisher, #29200) on a rotator at 4°C overnight. The bead‐sample complex was washed five times with 1 mL of ice‐cold HBSS by centrifuging (3000 × *g*, 5 min and 4°C). The samples were boiled to release the proteins for detection.

### 4.12 Co‐immunoprecipitation

The cells were lysed and harvested with RIPA buffer and kept on ice for 30 min. During this time, the Dynabeads Protein G and IP‐grade antibodies were rotated. Then, the cell lysis supernatant was collected and added to the antibody‐Dynabeads Protein G complex for 1.5 h under rotating conditions. The immunoprecipitants were washed and boiled for western blot analysis.

### 4.13 RNA‐FISH and immunofluorescence microscopy

An EPB41L4A‐AS1 RNA probe was labelled with Quasar 570 (Biosearch Technologies, SMF‐1063–5). Briefly, cells seeded on coverslips were washed, incubated with CSK buffer on ice, and fixed with 4% PFA. The cells were then dehydrated by treatment with 80, 95 and 100% ethanol for 3 min. Following this, the cells were incubated overnight with an EPB41L4A‐AS1 probe in a 2 × hybridization buffer at 37°C in a wet box. Next, 50% formamide and a 2 × SSC buffer were used to wash the cells at 42°C for 5 min three times. The cells were then blocked with BSA, treated with an IP‐grade GCN5 antibody (Abcam, ab217876), and finally treated with a secondary antibody. Last, the sections were counterstained with DAPI and observed under an Olympus FV1000 microscope.

### 4.14 Native RIP

Cells were harvested when they reached 90% confluence in 10 cm culture dishes and lysed with a polysome lysis buffer (PLB). The supernatants were incubated overnight with 5 μg of a GCN5 antibody (Abcam ab217876) or MLXIP antibody (Abcam, ab176688) and an IgG antibody (Abcam ab171870) at 4°C. Then, 80 μL of Dynabeads Protein G was added and rotated for 4 h. The bead‐protein‐RNA complex was washed with PLB containing 1 M urea. Next, RNA was precipitated overnight using ethanol, sodium acetate and LPA at −80°C. The RNA fraction was washed and purified with 75% ethanol and reverse transcribed. Target RNAs were analyzed by qRT‐PCR, each sample was calculated by 10% input and normalized by IgG. The primers (5′‐3′) used for detecting EPB41L4A‐AS1 enrichment were as follows: primer‐1, F: CCTGGTTTTAT TTTCGTCA and R: ATCCATCTTCCACCTGTAG‐3′; primer‐2, F: GTCAGCCAGTC AGCAACCT and R: ACTTAT GCAGCTCCTACACC.

### 4.15 RNA pull‐down assay

Cells were harvested when they reached 95% confluence, and the nuclear proteins were extracted. Different fragments of EPB41L4A‐AS1 plasmids were linearized by digestion with XhoI (Takara, 1635) and were transcribed using a MEGAscript kit (Ambion, 1334). Next, poly(A) was added to the RNAs using a poly(A) Tailing Kit 1 (Ambion, 1350). Each fragment anti‐sense RNA (or RNA without poly(A)) was used as a control. Approximately, 10 μg of poly(A)‐tailed RNAs was incubated with 100 μL of Oligo(dT)_25_ Dynabeads. The complex was incubated with a cell lysis buffer at 4°C for 2 h and the cell lysis buffer was used to wash the complex five times. The proteins were then released and detected.

### 4.16 Data analysis

Data are presented in terms of mean ± SD values, unless indicated otherwise. The western blot grey values were measured using ImageJ 1.50. Data plots were constructed by GraphPad Prism 8.0. Statistical significance was set at *p* < .05 (* represents *p* < .05, ** represents *p* < .01, *** represents *p* < .001 and ns represents *p* > .05). SPSS 20.0 and GraphPad Prism 8.0 were used to carry out statistical analysis with Mann–Whitney U test, two‐tailed Student's *t‐*test or one‐way analysis of variance, followed by Tukey's post‐hoc test.

## CONFLICT OF INTEREST

All the ten authors declared that there was no conflict of interest.

## Supporting information




**Supplementary Figure S1: EPB41L4A‐AS1 is upregulated in type 2 diabetes mellitus cell models**. (A‐B) Number of human primary skeletal muscle cells (HSkMC) and A673 cells treated at a high glucose concentration for 24 h (n = 3), as determined using the CCK‐8 assay. (C‐D) Number of L02 and HepG2 cells (n = 3), as determined using the CCK‐8 assay.

**Supplementary Figure S2: Glucose or glucosamine, at high concentrations, upregulates the expression of EPB41L4A‐AS1 by enhancing TP53 expression**. (A) Biological repeats of western blots related to Figure 2I. (B) Biological repeats of western blots related to Figure 2J. (C‐E) L02, HepG2, and A673 cells were transfected with NC, siP53 or siP53, and mutP53, and the P53 and mutP53 protein levels were measured. (F) Wild‐type and P53 knockout HeLa cells were treated at a high glucose concentration, and EAS1 expression was evaluated (n = 3). (G) HeLa and P53 knockout HeLa cells were induced with 5 mM glucosamine, and EAS1 expression was evaluated (n = 3).

**Supplementary Figure S3: EPB41L4A‐AS1 regulates glucose uptake**. As shown in the figure, EAS1 represents EPB41L4A‐AS1. (A) EPB41L4A‐AS1 expression in different normal tissues, based on data obtained from the GTEx database and the GTEXPORTAL website. (B) Schematic diagram of TIGA1 (green) and EPB41L4A‐AS1‐ATG‐mut. The bases shown in red represent the mutation sites. (C) The RNA over‐expression efficiency of EPB41L4A‐AS1 or EPB41L4A‐AS1‐ATG‐mut in HepG2, A673, and L02 cells was measured (n = 3). (D) The levels of TIGA1 protein in HepG2, A673, and L02 cells overexpressing EPB41L4A‐AS1 or EPB41L4A‐AS1‐ATG‐mut. (E) The efficiency of EPB41L4A‐AS1 knockdown in HepG2, A673, and L02 cells (n = 3). (F) EPB41L4A‐AS1 orthologue analysis using Multiz Alignments in the UCSC Genome Browser.

**Supplementary Figure S4: EPB41L4A‐AS1 regulates mitochondrial respiration**. As shown in the figure, EAS1 represents EPB41L4A‐AS1. (A‐C) The oxygen consumption rate (OCR) in cells treated at a high glucose concentration for 24 h after the transient overexpression of EPB41L4A‐AS1‐ATG‐mut, as measured using the Seahorse XFp assay. Basal respiration, proton leak, and maximal respiration were calculated (n = 3). (D‐F) The OCR was measured using the Seahorse XFp assay. Basal respiration, proton leakage, and maximal respiration were calculated (n = 3). (G‐I) Cell number after treatment at high glucose concentrations for 24 h (n = 3).

**Supplementary Figure S5: EPB41L4A‐AS1 regulates glucose uptake through GLUT2 or GLUT4**. As shown in the figure, EAS1 represents EPB41L4A‐AS1. (A‐B) Biological repeats related to Figure 4E‐F. (C‐D) Biological repeats related to Figure 4G‐H.

**Supplementary Figure S6: EPB41L4A‐AS1 negatively regulates GLUT4 transcription by increasing H3K27 crotonylation and PGC1β lysine acetylation**. As shown in the figure, EAS1 represents EPB41L4A‐AS1. (A) Biological repeats related to Figure 5G. (B) PGC1β and GCN5 levels in shNC and shEAS1 A673 cells, as measured by western blotting and qRT‐PCR (n = 3). (C) Biological repeats related to Figure S6B. (D‐E) Biological repeats related to Figure 5 M. (F‐G) Biological repeats related to Figure 5N. (H) Biological repeats related to Figure 5O‐Q.

**Supplementary Figure S7: EPB41L4A‐AS1 regulates H3K27cr via interaction with GCN5**. (A) Interaction between GCN5 and H3K27cr in L02 and HepG2 cells assayed by immunoprecipitation. (B) Biological repeats related to Figure 6 M.

**Supplementary Figure S8: EPB41L4A‐AS1 activates TXNIP transcription via the enhancement of H3K14 and H3K27 acetylation**. As shown in the figure, EAS1 represents EPB41L4A‐AS1; (A‐C) Biological repeats related to Figure 7E. (D) The histone mark H3K27ac across the sequence of TXNIP, based on data from the UCSC genome browser and the ENCODE database. (E‐F) H3K27ac enrichment in the TXNIP promoter in HepG2 and L02 cells (n = 3) assessed using chromatin immunoprecipitation (ChIP)‐qPCR analysis. (G‐H) H3K14ac occupation in the TXNIP promoter in HepG2 and L02 cells (n = 3) assessed using ChIP‐qPCR analysis. (I) Biological repeats related to Figure 7L.

FigureLegends
