# Supplementary figures and images for: Persistent high glucose induced EPB41L4A‐AS1 inhibits glucose uptake via GCN5 mediating crotonylation and acetylation of histones and non‐histones
Source: Clin Transl Med. 2022 Feb 20;12(2):e699. doi: 10.1002/ctm2.699 (PMC8858623; doi:10.1002/ctm2.699)

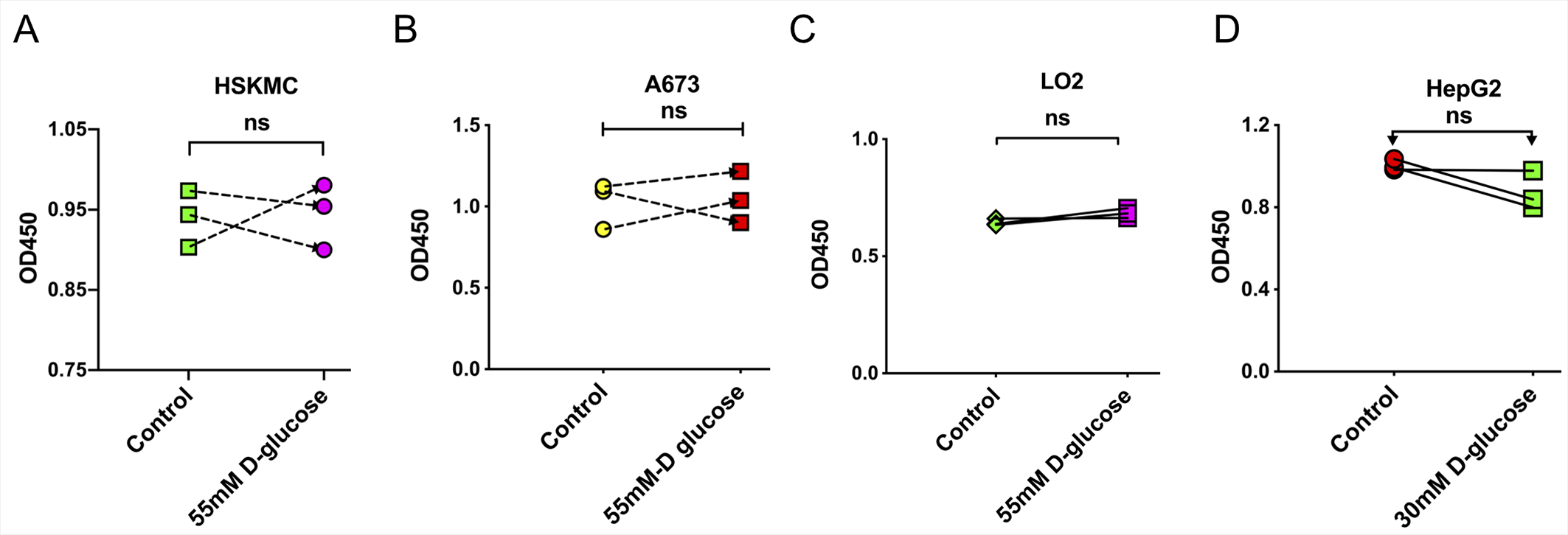

Supplement: Supplementary file 1 — Supplementary Figure S1: EPB41L4A‐AS1 is upregulated in type 2 diabetes mellitus cell models. (A‐B) Number of human primary skeletal muscle cells (HSkMC) and A673 cells treated at a high glucose concentration for 24 h (n = 3), as determined using the CCK‐8 assay. (C‐D) Number of L02 and HepG2 cells (n = 3), as determined using the CCK‐8 assay. [file CTM2-12-e699-s003.tiff]

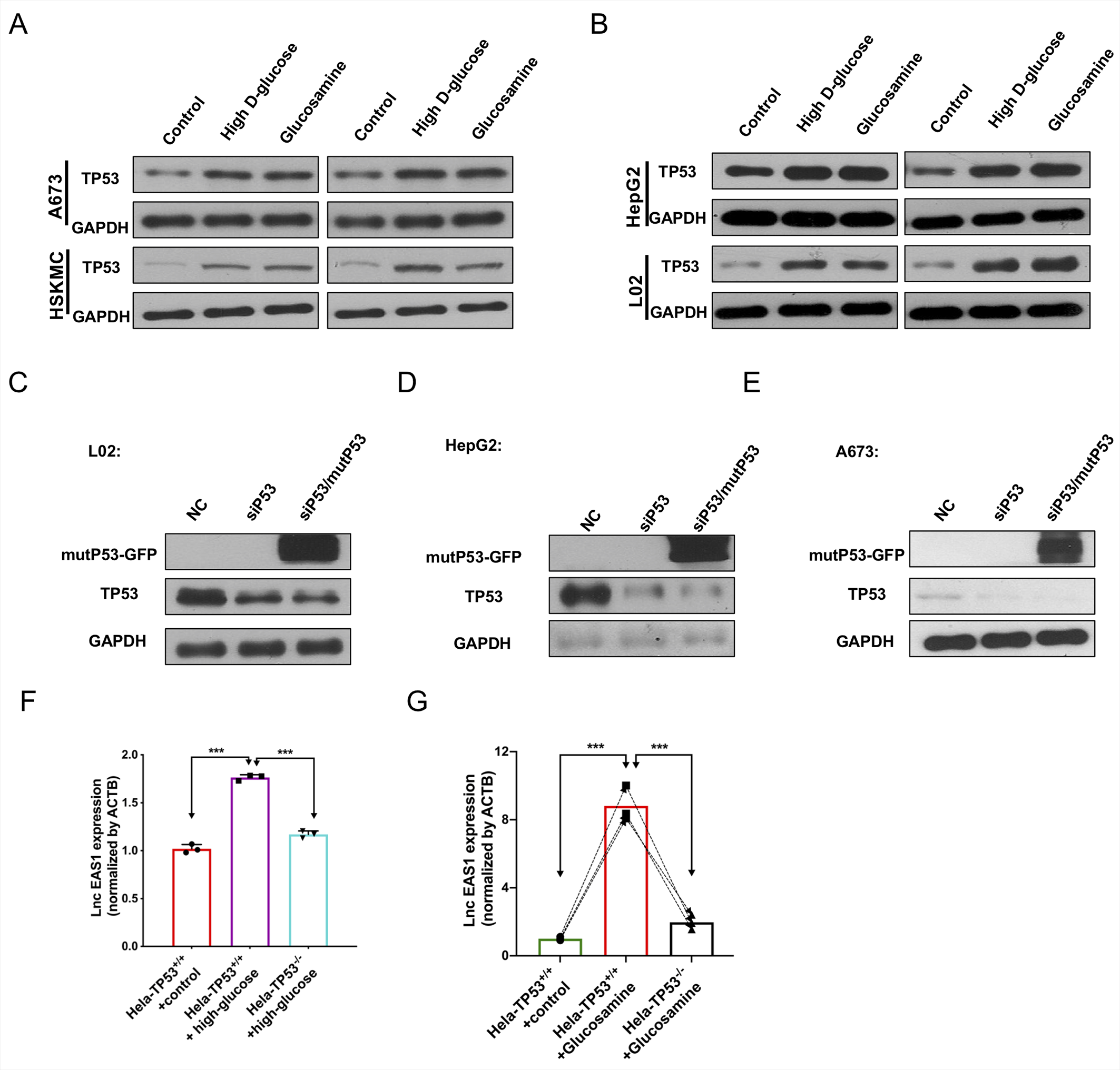

Supplement: Supplementary file 2 — Supplementary Figure S2: Glucose or glucosamine, at high concentrations, upregulates the expression of EPB41L4A‐AS1 by enhancing TP53 expression. (A) Biological repeats of western blots related to Figure 2I. (B) Biological repeats of western blots related to Figure 2J. (C‐E) L02, HepG2, and A673 cells were transfected with NC, siP53 or siP53, and mutP53, and the P53 and mutP53 protein levels were measured. (F) Wild‐type and P53 knockout HeLa cells were treated at a high glucose concentration, and EAS1 expression was evaluated (n = 3). (G) HeLa and P53 knockout HeLa cells were induced with 5 mM glucosamine, and EAS1 expression was evaluated (n = 3). [file CTM2-12-e699-s005.tiff]

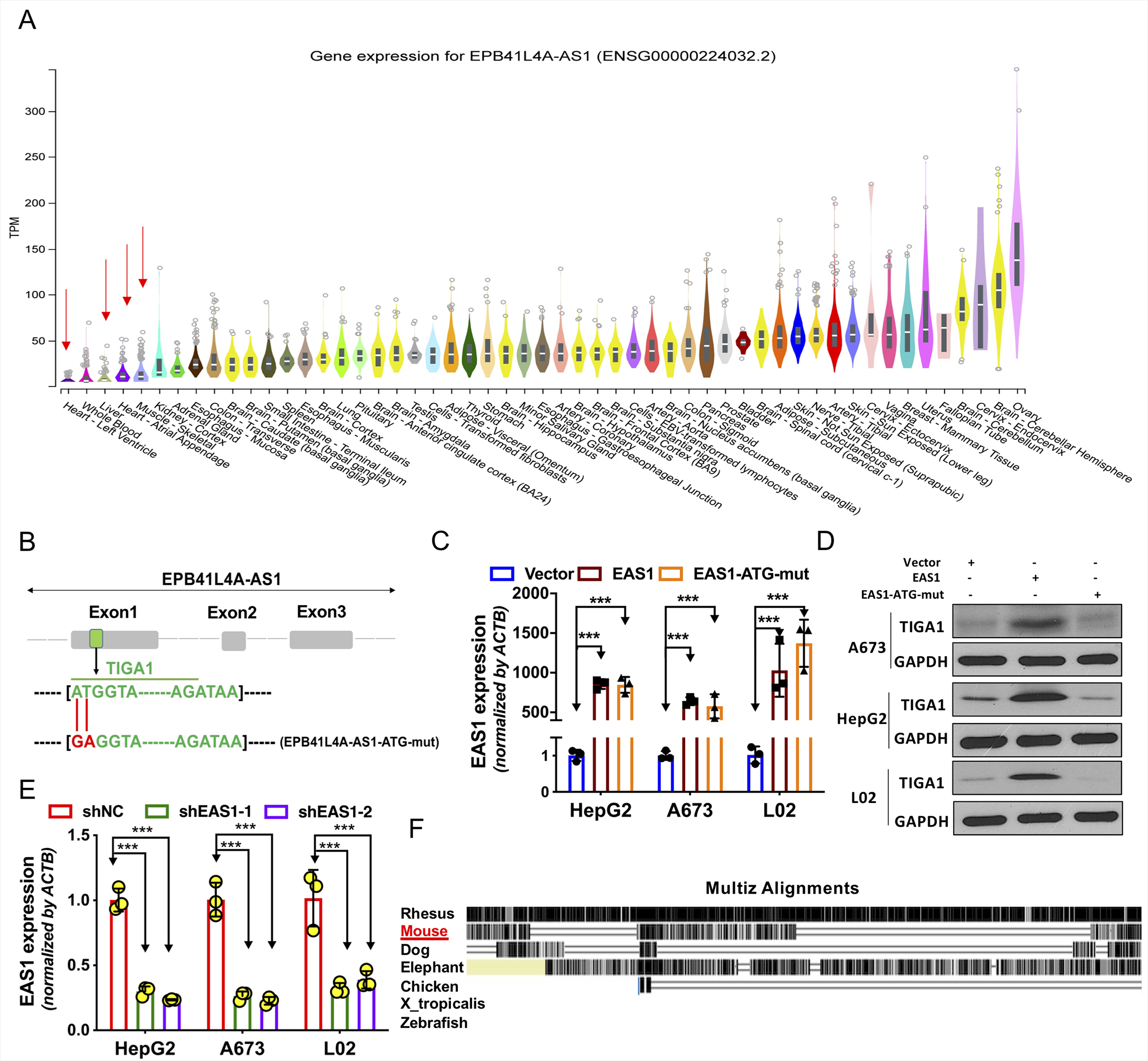

Supplement: Supplementary file 3 — Supplementary Figure S3: EPB41L4A‐AS1 regulates glucose uptake. As shown in the figure, EAS1 represents EPB41L4A‐AS1. (A) EPB41L4A‐AS1 expression in different normal tissues, based on data obtained from the GTEx database and the GTEXPORTAL website. (B) Schematic diagram of TIGA1 (green) and EPB41L4A‐AS1‐ATG‐mut. The bases shown in red represent the mutation sites. (C) The RNA over‐expression efficiency of EPB41L4A‐AS1 or EPB41L4A‐AS1‐ATG‐mut in HepG2, A673, and L02 cells was measured (n = 3). (D) The levels of TIGA1 protein in HepG2, A673, and L02 cells overexpressing EPB41L4A‐AS1 or EPB41L4A‐AS1‐ATG‐mut. (E) The efficiency of EPB41L4A‐AS1 knockdown in HepG2, A673, and L02 cells (n = 3). (F) EPB41L4A‐AS1 orthologue analysis using Multiz Alignments in the UCSC Genome Browser. [file CTM2-12-e699-s008.tiff]

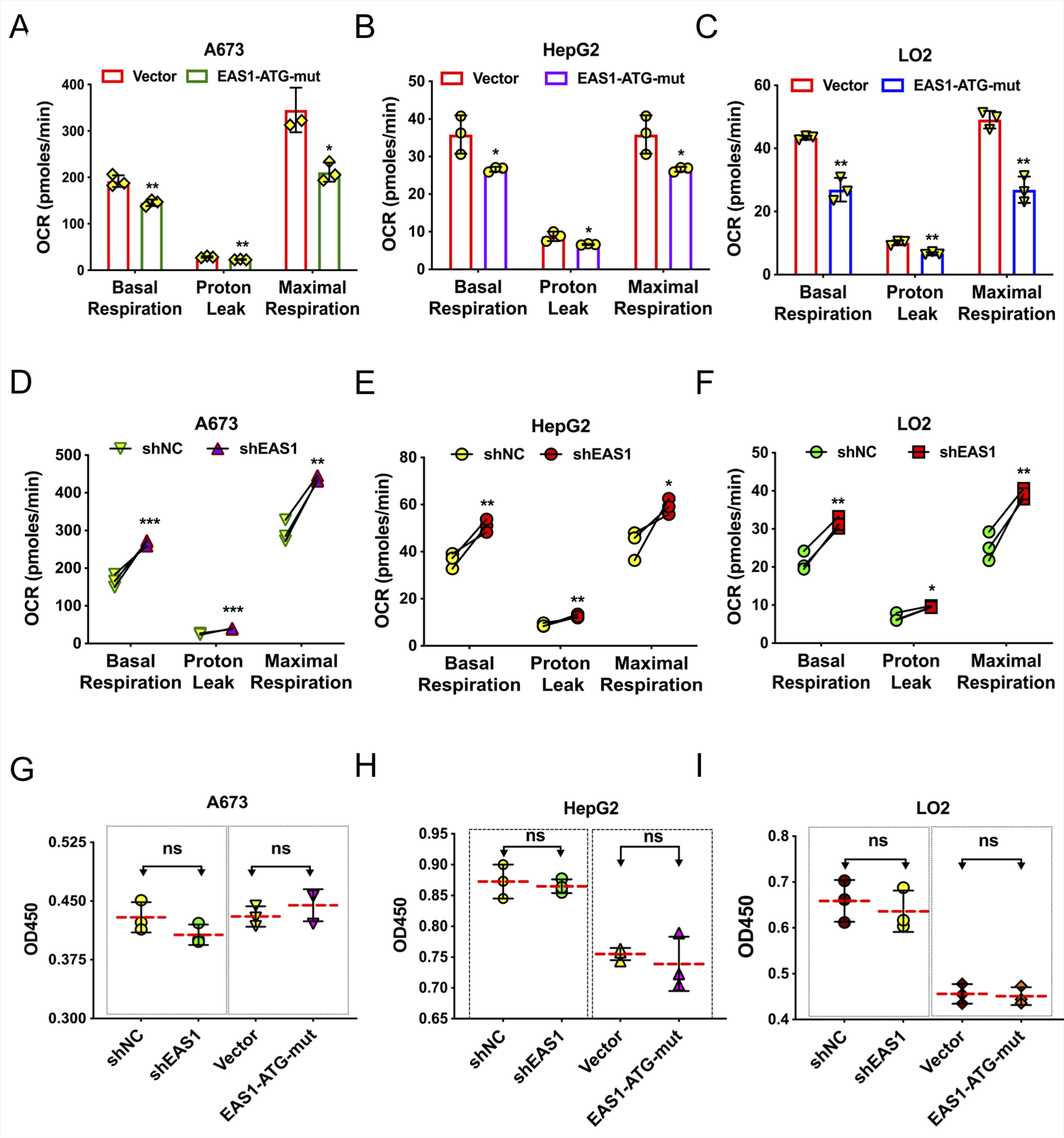

Supplement: Supplementary file 4 — Supplementary Figure S4: EPB41L4A‐AS1 regulates mitochondrial respiration. As shown in the figure, EAS1 represents EPB41L4A‐AS1. (A‐C) The oxygen consumption rate (OCR) in cells treated at a high glucose concentration for 24 h after the transient overexpression of EPB41L4A‐AS1‐ATG‐mut, as measured using the Seahorse XFp assay. Basal respiration, proton leak, and maximal respiration were calculated (n = 3). (D‐F) The OCR was measured using the Seahorse XFp assay. Basal respiration, proton leakage, and maximal respiration were calculated (n = 3). (G‐I) Cell number after treatment at high glucose concentrations for 24 h (n = 3). [file CTM2-12-e699-s009.tiff]

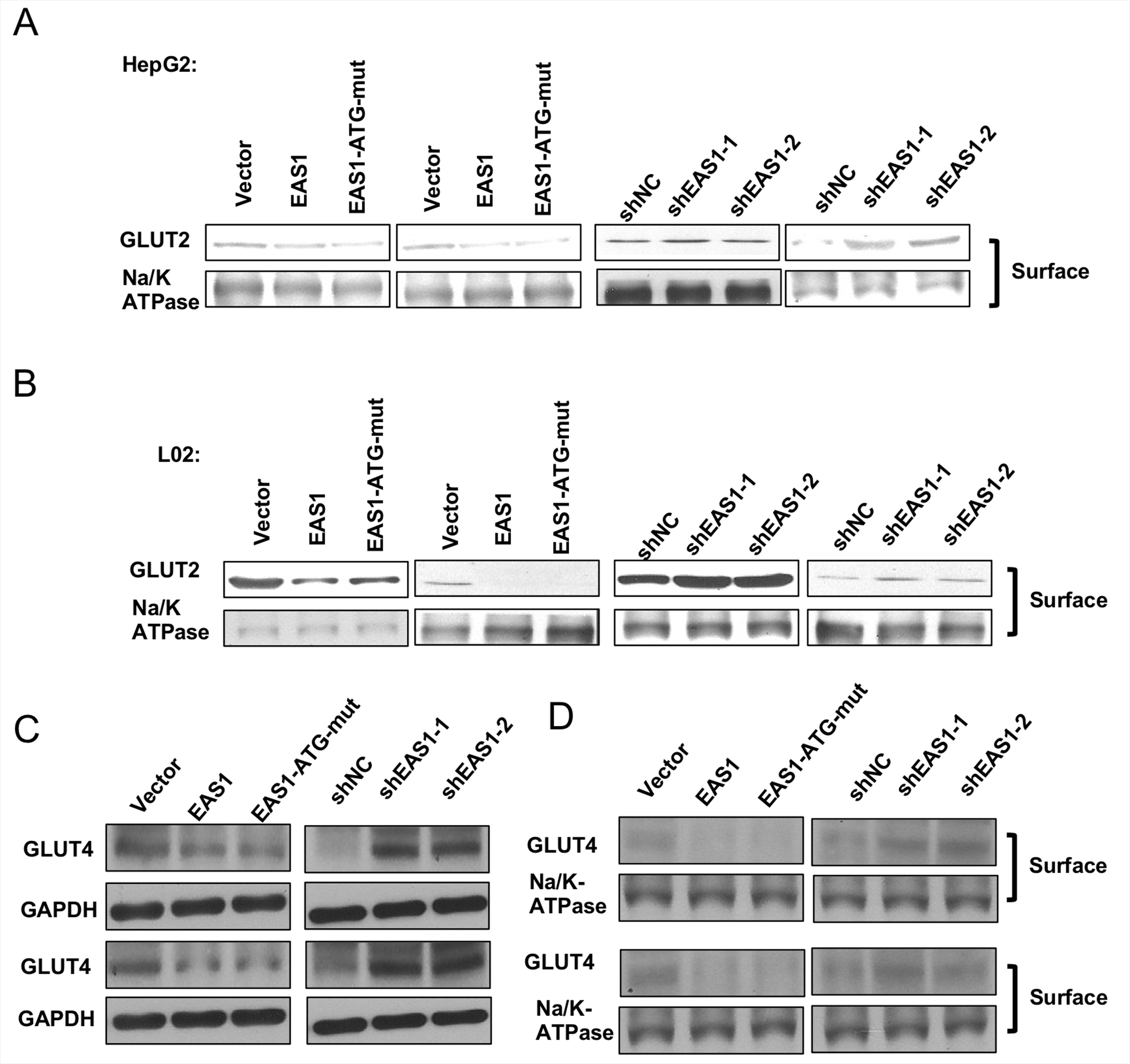

Supplement: Supplementary file 5 — Supplementary Figure S5: EPB41L4A‐AS1 regulates glucose uptake through GLUT2 or GLUT4. As shown in the figure, EAS1 represents EPB41L4A‐AS1. (A‐B) Biological repeats related to Figure 4E‐F. (C‐D) Biological repeats related to Figure 4G‐H. [file CTM2-12-e699-s002.tiff]

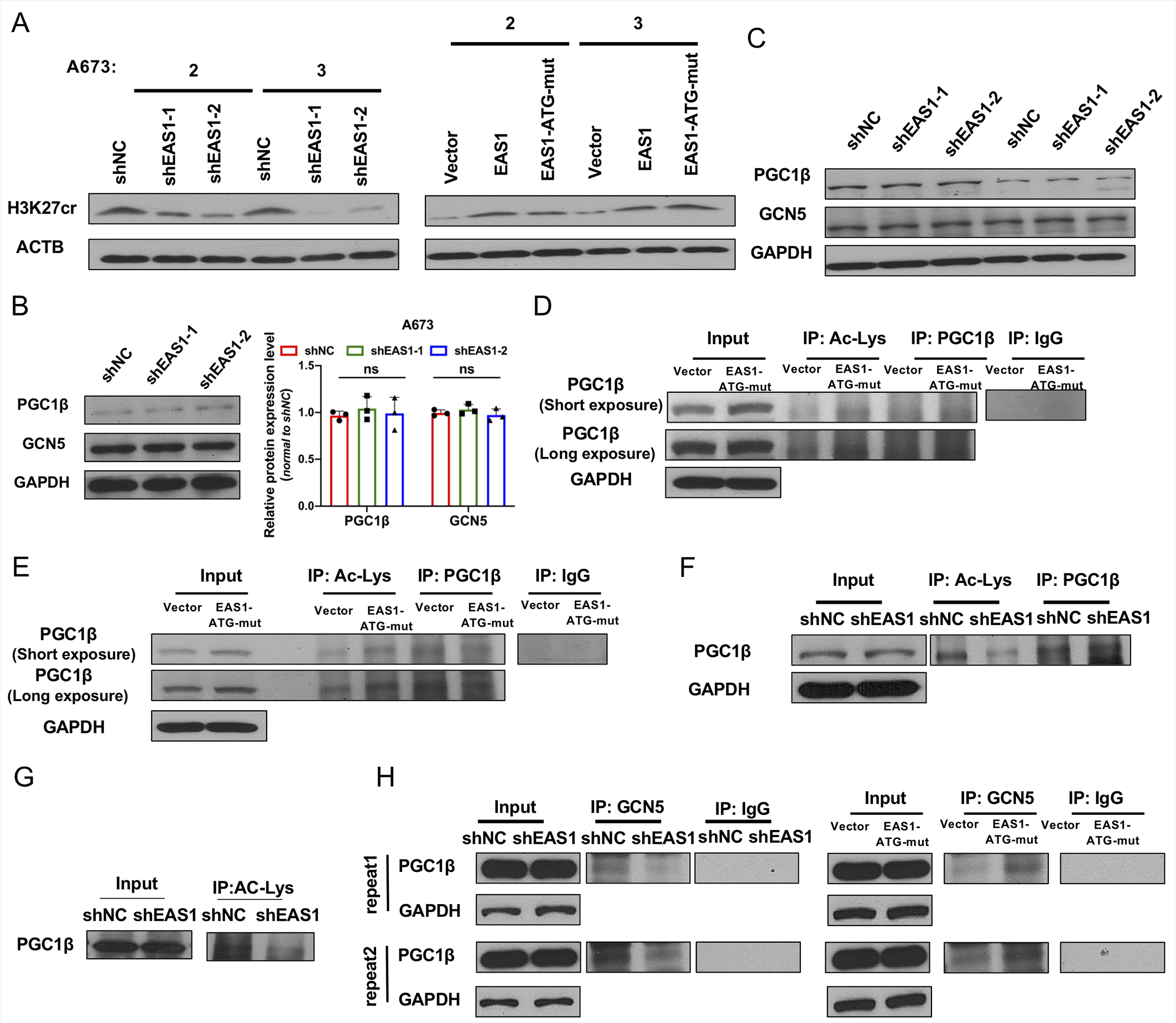

Supplement: Supplementary file 6 — Supplementary Figure S6: EPB41L4A‐AS1 negatively regulates GLUT4 transcription by increasing H3K27 crotonylation and PGC1β lysine acetylation. As shown in the figure, EAS1 represents EPB41L4A‐AS1. (A) Biological repeats related to Figure 5G. (B) PGC1β and GCN5 levels in shNC and shEAS1 A673 cells, as measured by western blotting and qRT‐PCR (n = 3). (C) Biological repeats related to Figure S6B. (D‐E) Biological repeats related to Figure 5 M. (F‐G) Biological repeats related to Figure 5N. (H) Biological repeats related to Figure 5O‐Q. [file CTM2-12-e699-s006.tiff]

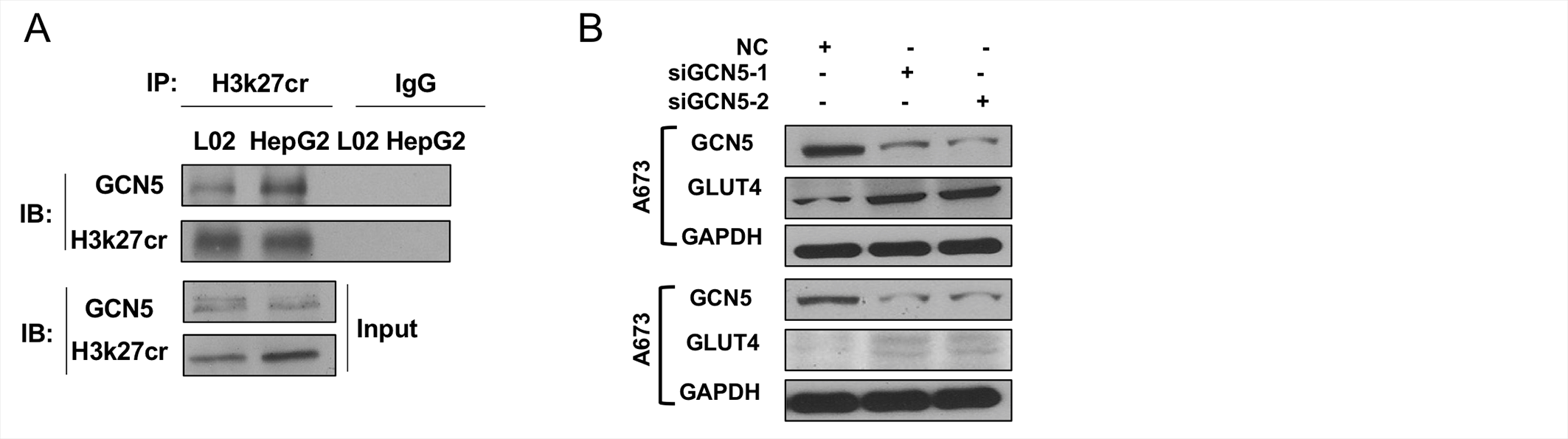

Supplement: Supplementary file 7 — Supplementary Figure S7: EPB41L4A‐AS1 regulates H3K27cr via interaction with GCN5. (A) Interaction between GCN5 and H3K27cr in L02 and HepG2 cells assayed by immunoprecipitation. (B) Biological repeats related to Figure 6 M. [file CTM2-12-e699-s004.tiff]

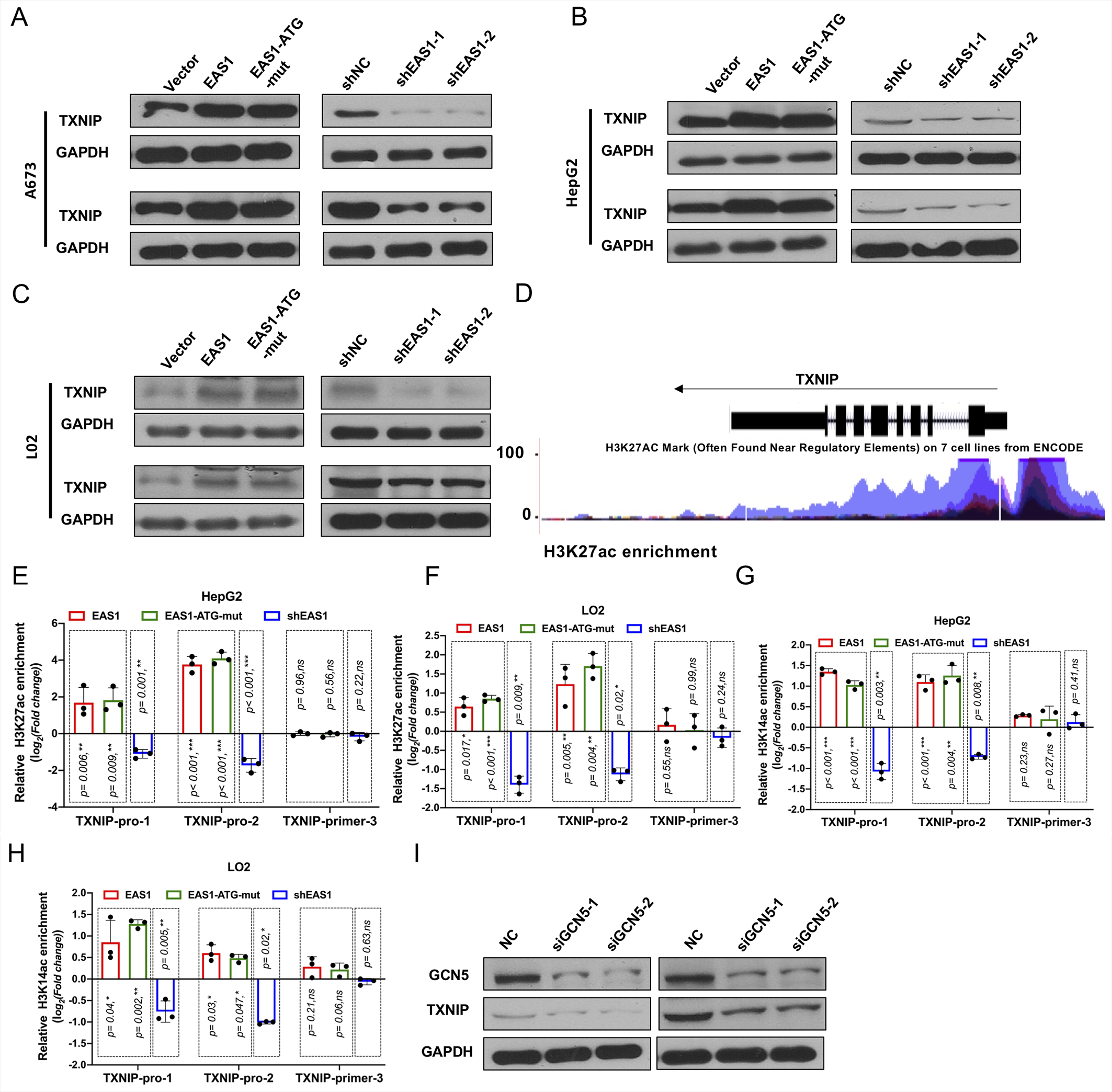

Supplement: Supplementary file 8 — Supplementary Figure S8: EPB41L4A‐AS1 activates TXNIP transcription via the enhancement of H3K14 and H3K27 acetylation. As shown in the figure, EAS1 represents EPB41L4A‐AS1; (A‐C) Biological repeats related to Figure 7E. (D) The histone mark H3K27ac across the sequence of TXNIP, based on data from the UCSC genome browser and the ENCODE database. (E‐F) H3K27ac enrichment in the TXNIP promoter in HepG2 and L02 cells (n = 3) assessed using chromatin immunoprecipitation (ChIP)‐qPCR analysis. (G‐H) H3K14ac occupation in the TXNIP promoter in HepG2 and L02 cells (n = 3) assessed using ChIP‐qPCR analysis. (I) Biological repeats related to Figure 7L. [file CTM2-12-e699-s001.tif]
