# Supplementary material for: Persistent high glucose induced EPB41L4A‐AS1 inhibits glucose uptake via GCN5 mediating crotonylation and acetylation of histones and non‐histones
Source: Clin Transl Med. 2022 Feb 20;12(2):e699. doi: 10.1002/ctm2.699 (PMC8858623; doi:10.1002/ctm2.699)
Supplement: Supplementary file 9 — FigureLegends [file CTM2-12-e699-s007.docx]

**Supplementary Figure legends**

**Supplementary Figure S1: EPB41L4A-AS1 is upregulated in type 2 diabetes mellitus cell models.** (A-B) Number of human primary skeletal muscle cells (HSkMC) and A673 cells treated at a high glucose concentration for 24 h (n = 3), as determined using the CCK-8 assay. (C-D) Number of L02 and HepG2 cells (n = 3), as determined using the CCK-8 assay.

**Supplementary Figure S2:** **Glucose or glucosamine, at high concentrations, upregulates the expression of EPB41L4A-AS1 by enhancing TP53 expression.** (A) Biological repeats of western blots related to Figure 2I. (B) Biological repeats of western blots related to Figure 2J. (C-E) L02, HepG2, and A673 cells were transfected with NC, siP53 or siP53, and mutP53, and the P53 and mutP53 protein levels were measured. (F) Wild-type and P53 knockout HeLa cells were treated at a high glucose concentration, and EAS1 expression was evaluated (n = 3). (G) HeLa and P53 knockout HeLa cells were induced with 5 mM glucosamine, and EAS1 expression was evaluated (n = 3).

**Supplementary Figure S3: EPB41L4A-AS1 regulates glucose uptake.** As shown in the figure, EAS1 represents EPB41L4A-AS1. (A) EPB41L4A-AS1 expression in different normal tissues, based on data obtained from the GTEx database and the GTEXPORTAL website. (B) Schematic diagram of TIGA1 (green) and EPB41L4A-AS1-ATG-mut. The bases shown in red represent the mutation sites. (C) The RNA overexpression efficiency of EPB41L4A-AS1 or EPB41L4A-AS1-ATG-mut in HepG2, A673, and L02 cells was measured (n = 3). (D) The levels of TIGA1 protein in HepG2, A673, and L02 cells overexpressing EPB41L4A-AS1 or EPB41L4A-AS1-ATG-mut. (E) The efficiency of EPB41L4A-AS1 knockdown in HepG2, A673, and L02 cells (n = 3). (F) EPB41L4A-AS1 orthologue analysis using Multiz Alignments in the UCSC Genome Browser.

**Supplementary Figure S4: EPB41L4A-AS1 regulates mitochondrial respiration.** As shown in the figure, EAS1 represents EPB41L4A-AS1. (A-C) The oxygen consumption rate (OCR) in cells treated at a high glucose concentration for 24 h after the transient overexpression of EPB41L4A-AS1-ATG-mut, as measured using the Seahorse XFp assay. Basal respiration, proton leak, and maximal respiration were calculated (n = 3). (D-F) The OCR was measured using the Seahorse XFp assay. Basal respiration, proton leakage, and maximal respiration were calculated (n = 3). (G-I) Cell number after treatment at high glucose concentrations for 24 h (n = 3).

**Supplementary Figure S5: EPB41L4A-AS1 regulates glucose uptake through GLUT2 or GLUT4.** As shown in the figure, EAS1 represents EPB41L4A-AS1. (A-B) Biological repeats related to Figure 4E-F. (C-D) Biological repeats related to Figure 4G-H.

**Supplementary Figure S6: EPB41L4A-AS1 negatively regulates GLUT4 transcription by increasing H3K27 crotonylation and PGC1β lysine acetylation.** As shown in the figure, EAS1 represents EPB41L4A-AS1. (A) Biological repeats related to Figure 5G. (B) PGC1β and GCN5 levels in shNC and shEAS1 A673 cells, as measured by western blotting and qRT-PCR (n = 3). (C) Biological repeats related to Figure S6B. (D-E) Biological repeats related to Figure 5M. (F-G) Biological repeats related to Figure 5N. (H) Biological repeats related to Figure 5O-Q.

**Supplementary Figure S7: EPB41L4A-AS1 regulates H3K27cr via interaction with GCN5.** (A) Interaction between GCN5 and H3K27cr in L02 and HepG2 cells assayed by immunoprecipitation. (B) Biological repeats related to Figure 6M.

**Supplementary Figure S8: EPB41L4A-AS1 activates TXNIP transcription via the enhancement of H3K14 and H3K27 acetylation.** As shown in the figure, EAS1 represents EPB41L4A-AS1; (A-C) Biological repeats related to Figure 7E. (D) The histone mark H3K27ac across the sequence of TXNIP, based on data from the UCSC genome browser and the ENCODE database. (E-F) H3K27ac enrichment in the TXNIP promoter in HepG2 and L02 cells (n = 3) assessed using chromatin immunoprecipitation (ChIP)-qPCR analysis. (G-H) H3K14ac occupation in the TXNIP promoter in HepG2 and L02 cells (n = 3) assessed using ChIP-qPCR analysis. (I) Biological repeats related to Figure 7L.
